# Rise in broadly cross-reactive adaptive immunity against human β-coronaviruses in MERS-recovered patients during the COVID-19 pandemic

**DOI:** 10.1126/sciadv.adk6425

**Published:** 2024-02-28

**Authors:** So-Hee Kim, Yuri Kim, Sangeun Jeon, Uni Park, Ju-Il Kang, Kyeongseok Jeon, Hye-Ran Kim, Songhyeok Oh, Ji-Young Rhee, Jae-Phil Choi, Wan Beom Park, Sang Won Park, Jeong-Sun Yang, Joo-Yeon Lee, Jihye Kang, Hyoung-Shik Shin, Yeonjae Kim, Seungtaek Kim, Yeon-Sook Kim, Dong-Gyun Lim, Nam-Hyuk Cho

**Affiliations:** ^1^ Department of Microbiology and Immunology, College of Medicine, Seoul National University, Seoul 03080, Republic of Korea.; ^2^ Department of Biomedical Sciences, College of Medicine, Seoul National University, Seoul 03080, Republic of Korea.; ^3^ Institute of Endemic Disease, Seoul National University Medical Research, Seoul 03080, Republic of Korea.; ^4^ Zoonotic Virus Laboratory, Institut Pasteur Korea, Seongnam 13488, Republic of Korea.; ^5^ Division of Infectious Diseases, Department of Medicine, Dankook University College of Medicine, Cheonan 31116, Republic of Korea.; ^6^ Department of Internal Medicine, Seoul Medical Center, Seoul 02053, Republic of Korea.; ^7^ Department of Internal Medicine, Seoul National University College of Medicine, Seoul 03080, Republic of Korea.; ^8^ Center for Emerging Virus Research, Korea National Institute of Health, Korea Disease Control and Prevention Agency, Cheongju 28159, Republic of Korea.; ^9^ Translational Research Center, Research Institute of Public Health, National Medical Center, Seoul 04564, Republic of Korea.; ^10^ Division of Infectious Diseases, Department of Internal Medicine, Daejeon Eulji Medical Center, Eulji University School of Medicine, Daejeon 34824, Republic of Korea.; ^11^ Center for Infectious Diseases, National Medical Center, Seoul 04564, Republic of Korea.; ^12^ Division of Infectious Diseases, Department of Internal Medicine, Chungnam National University School of Medicine, Daejeon 35015, Republic of Korea.; ^13^ Seoul National University Bundang Hospital, Seongnam, Gyeonggi-do 13620, Republic of Korea.

## Abstract

To develop a universal coronavirus (CoV) vaccine, long-term immunity against multiple CoVs, including severe acute respiratory syndrome coronavirus 2 (SARS-CoV-2) variants, Middle East respiratory syndrome (MERS)–CoV, and future CoV strains, is crucial. Following the 2015 Korean MERS outbreak, we conducted a long-term follow-up study and found that although neutralizing antibodies and memory T cells against MERS-CoV declined over 5 years, some recovered patients exhibited increased antibody levels during the COVID-19 pandemic. This likely resulted from cross-reactive immunity induced by SARS-CoV-2 vaccines or infections. A significant correlation in antibody responses across various CoVs indicates shared immunogenic epitopes. Two epitopes—the spike protein’s stem helix and intracellular domain—were highly immunogenic after MERS-CoV infection and after SARS-CoV-2 vaccination or infection. In addition, memory T cell responses, especially polyfunctional CD4^+^ T cells, were enhanced during the pandemic, correlating significantly with MERS-CoV spike-specific antibodies and neutralizing activity. Therefore, incorporating these cross-reactive and immunogenic epitopes into pan-CoV vaccine formulations may facilitate effective vaccine development.

## Teaser

Antibody responses against various human coronaviruses in MERS-recovered patients define immunogenic and cross-reactive epitopes.

## INTRODUCTION

Severe acute respiratory syndrome coronavirus 2 (SARS-CoV-2), the causative agent of COVID-19, belongs to the family of positive-sense RNA human coronaviruses (hCoVs) (*1*). This family includes α-coronaviruses (hCoV-229E and hCoV-NL63) and β-coronaviruses [hCoV-HKU1, hCoV-OC43, Middle East respiratory syndrome coronavirus (MERS-CoV), and SARS-CoV]. All hCoVs have homologous genomic structures encoding the viral spike (S), envelope (E), membrane (M), and nucleocapsid (N) structural proteins; a large polymerase complex composed of 16 nonstructural proteins (NSPs); and several accessory proteins (*2*). These proteins retain highly conserved motifs among the various hCoVs, which may broadly support cross-reactive immune responses (*3*). Such cross-reactive responses to SARS-CoV-2 proteins preexist in naïve participants even before the COVID-19 pandemic, and most cross-reactive epitopes are located within the conserved structural proteins and NSPs of hCoVs (*3*–*7*). However, the specific role of these cross-reactive responses in the outcomes of SARS-CoV-2 infection and COVID-19 vaccination remains unclear (*3*). Nonetheless, hCoV cross-reactive antibodies are boosted by SARS-CoV-2 infection (*8*). Considering that repeated infections with different SARS-CoV-2 variants and other hCoVs, along with COVID-19 vaccination, may have continued in the post–COVID-19 pandemic era, cross-reactive memory responses against various hCoVs may form a complex pool of heterogeneous herd immunity against the valid viral pathogens. Therefore, the specific role of broad cross-reactivity against various hCoVs needs to be determined to prevent severe disease progression and to develop effective and universal hCoV vaccines for future CoV pandemics.

Newly emerging zoonotic CoVs that cause acute respiratory syndrome have threatened global public health via potential spillovers from animal hosts, such as bats (*9*). SARS-CoV and MERS-CoV emerged in 2002 and 2012, respectively, whereas SARS-CoV-2 has spread globally at an alarming rate since December 2019 and has successfully adapted to the human population. Although their transmission potential and virulence in humans vary greatly, the continuous challenge of diverse zoonotic CoVs may create a unique immune repertoire against the CoV family in humans. However, our current knowledge of the long-term dynamics and impact of managing the repeated challenge of various CoVs on the human immune system is limited. In addition, a recent global vaccine campaign against COVID-19 has introduced an additional layer of artificial immune modulation against the viral pathogens. In South Korea, the confirmed SARS-CoV-2 infection rate was 1.2% of the entire population at the end of 2021, which increased to 56.3% by the end of 2022. In addition, COVID-19 vaccine coverage expanded to 85.0% in 2021 and further to 86.7% by the end of 2022 (https://covid19.who.int/). Therefore, tracing adaptive immunity in humans could elucidate the mechanistic understanding of long-term changes in our immune system against ongoing insults by SARS-CoV-2 variants, sporadic MERS-CoV outbreaks, and future novel pandemic CoVs.

In 2015, a large MERS outbreak swept South Korea, resulting in 186 confirmed cases and 38 deaths (*10*). This outbreak was initiated by an infected traveler from the Middle East and amplified in healthcare settings (*11*). Our group launched a Korean MERS cohort in 2016 and performed follow-up studies for up to 7 years, including the COVID-19 pandemic period, to evaluate MERS-CoV–specific adaptive immune responses in MERS-recovered patients (*10*, *12*–*16*). The present study aimed to further explore the antibody responses against hCoVs, including SARS-CoV, SARS-CoV-2, and MERS-CoV. This enabled us to landscape the chronological changes in adaptive immunity against various hCoVs in MERS-recovered patients during the COVID-19 pandemic and highlight the heterogeneous but focused boosting of cross-reactive antibody responses, as well as neutralizing activity, against conserved epitopes shared by diverse hCoVs, including SARS-CoV and SARS-CoV-2.

## RESULTS

### Increased neutralizing antibody levels against MERS-CoV in MERS-recovered patients during the COVID-19 pandemic

According to disease severity during the 2015 MERS outbreak in Korea (*13*), the participants were classified into group I (G I): asymptomatic or those with mild disease not progressing to pneumonia; group II (G II): those with mild pneumonia without hypoxemia; and group III (G III): those recovering from prolonged and severe pneumonia, where they experienced hypoxemia and were treated with oxygen during hospitalization (table S1).

**Fig. 1.**
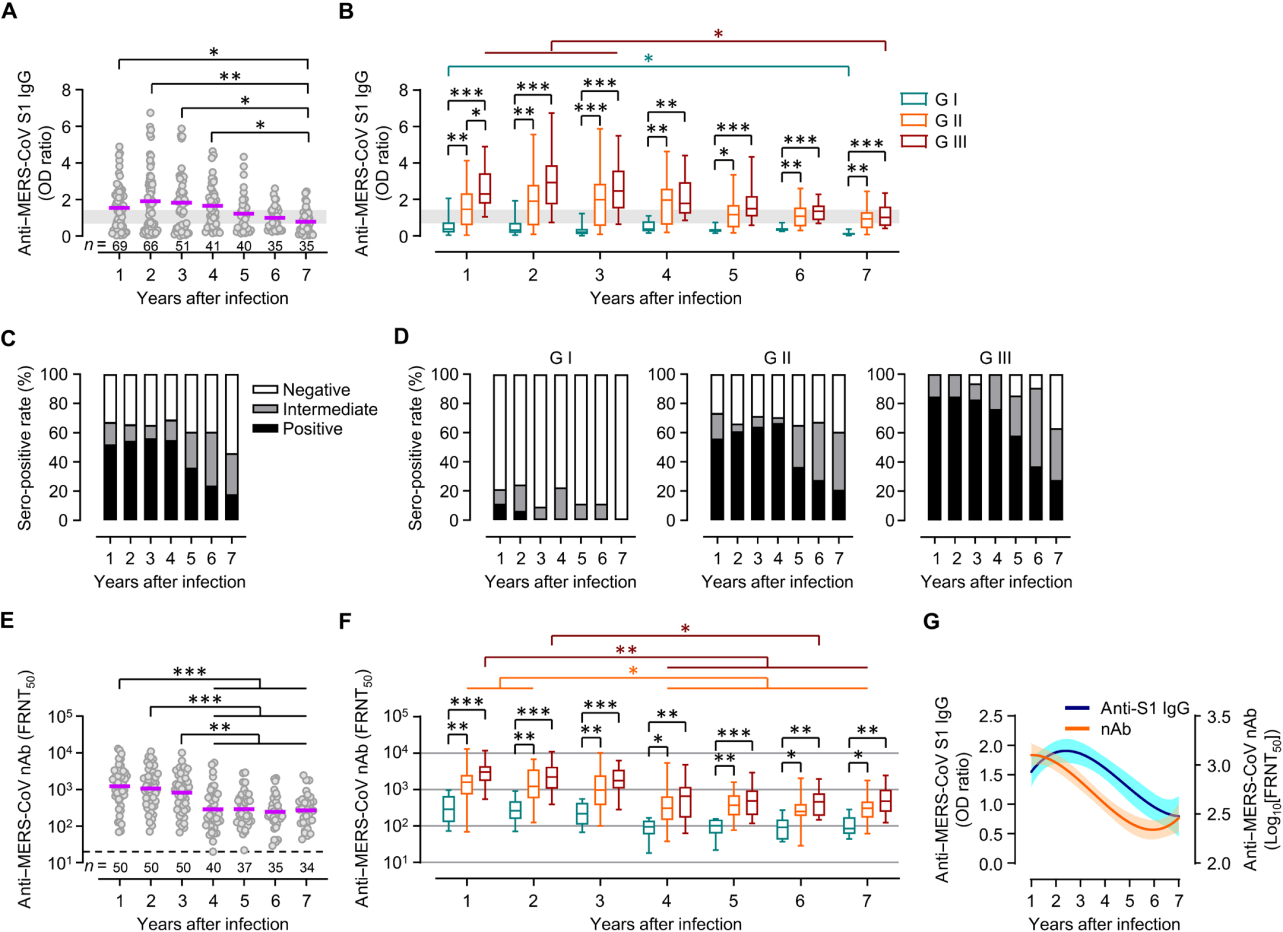
Kinetic changes in S1 IgG antibody responses, seropositivity, and neutralizing activity against MERS-CoV in recovered patients. (**A**) The serum spike (S1)–specific IgG levels were semiquantitatively determined by calculating the OD ratios. Intermediate OD ratio range is marked gray. (**B**) Anti-S1 lgG levels according to clinical MERS severity represented as box and whisker (minimum to maximum) plots, including the median value. (**C** and **D**) Seropositivity based on OD ratios in all participants (C) and in each clinical severity group (D) at each time point. (**E**) Neutralizing activity (FRNT_50_) levels of collected sera. Dashed line: Limit of detection. (**F**) Neutralizing activities according to clinical MERS severity. (**G**) Kinetic changes in mean OD ratios against the S1 antigen and neutralizing activity after nonlinear regression analysis (solid line) with 95% confidence intervals (CIs) (shaded cyan and orange). nAb, neutralizing antibody; *n*, sample number; purple line, geometric mean value; *P < 0.05; **P < 0.01; ***P < 0.001 by Kruskal-Wallis test.

Specific antibody responses against the MERS-CoV spike antigen (S1) were assessed in serum samples collected from participants up to 7 years after symptom onset (Fig. 1A). The mean optical density (OD) ratios against S1 peaked at the second year (mean ± SD, 1.90 ± 1.69) and gradually decreased thereafter (0.78 ± 0.67 at the seventh year). The magnitude and durability of antibody responses and the seropositivity rate strongly correlated with disease severity. Patients in G II and G III showed stronger and sustained antibody responses than those in G I (Fig. 1B). The seropositivity rate persisted for up to the fourth year (50.7 to 54.9%) and sharply decreased to 17.1% in the seventh year (Fig. 1, C and D). No participant in G I was seropositive from the third year, whereas 75.0 to 83.3% of the participants in G III showed persistent seroconversion up to the fourth year, and 27.3% of the participants remained seropositive in the seventh year. The foci reduction neutralization titer (FRNT_50_) against MERS-CoV persisted up to the third year and significantly decreased from the fourth year after infection (Fig. 1E). The mean FRNT_50_ value in the first year (mean ± SD, 2359 ± 2813) was reduced by 70.4% in the fourth year (699 ± 1187) and by 82.1% in the sixth year (422 ± 511). The mean neutralizing antibody (nAb) titer marginally increased in the seventh year (450 ± 543, 6.7% increase relative to that in the sixth year) in contrast to the continuously reducing antibody levels against the S1 antigen (Fig. 1G). Although the nAb titers were generally higher among patients in G II and G III than those in participants of G I throughout the follow-up period (Fig. 1F), the mean FRNT_50_ values in all three groups were consistently elevated in the seventh year (percent increase: G I = 8.6%, G II = 3.8%, and G III = 13.0%) compared to those in the sixth year. Thus, the neutralizing activity against MERS-CoV in our cohort may have been affected by active vaccination campaigns and/or SARS-CoV-2 infection in South Korea during the COVID-19 pandemic.

### Increased cross-reacting antibody levels against human βCoVs in MERS-recovered patients during the COVID-19 pandemic

**Fig. 2.**
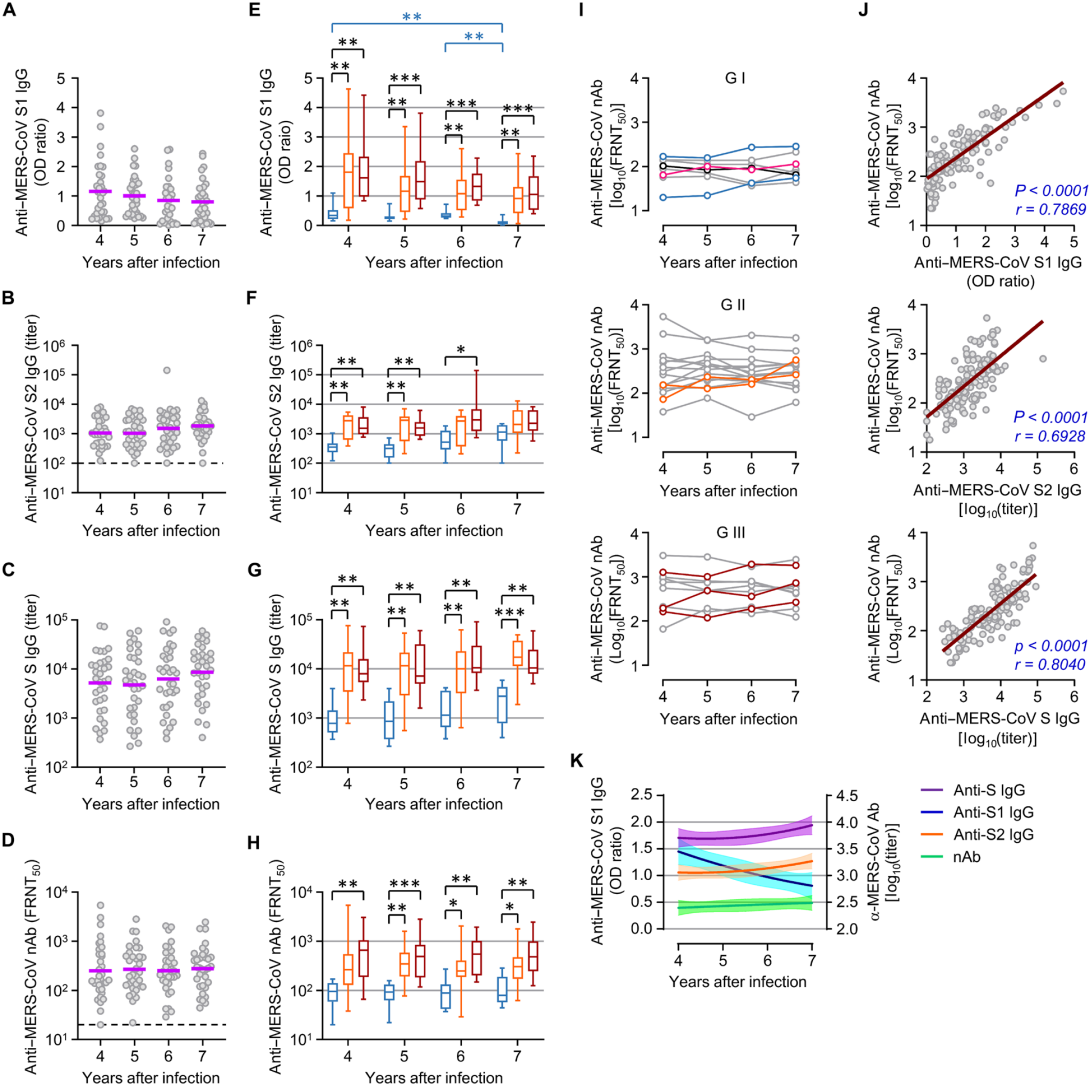
Kinetic changes in antibody responses against spike antigens and neutralizing activity against MERS-CoV. (**A** to **D**) Antibody response levels against indicated MERS-CoV spike antigens and neutralizing activity 4 to 7 years after the Korean MERS outbreak. Purple line, geometric mean; nAb, neutralizing antibody; dashed line, limit of detection; *n* = 33 per year. (**E** to **H**) The antibody response levels according to clinical MERS severity represented as box and whisker (minimum to maximum) plots, including the median value. *n* = 33 per year. (**I**) Kinetic changes in individual neutralizing activities with indicated disease severity. Kinetic lines of seven participants with >50% increase in neutralizing activity during the COVID-19 pandemic, according to MERS severity (light blue: G I, orange: G II, brown: G III, pink: unvaccinated and SARS-CoV-2–infected participant, and black: unvaccinated and uninfected participant). (**J**) Correlation of neutralizing activity against MERS-CoV with the indicated antibody levels were assessed using linear regression (brown line) and Spearman’s rank test (*r* and *P* value in indigo). *n* = 132 (33 × 4 years). (**K**) Kinetic changes in mean antibody levels against the indicated spike antigens and neutralizing activity after nonlinear regression analysis (solid line) with 95% CI (shaded colors). *P < 0.05; **P < 0.01; ***P < 0.001 using the Kruskal-Wallis test.

To investigate the sudden increase in nAb levels against MERS-CoV in the seventh year, we further investigated the kinetic changes in specific antibody responses against MERS-CoV spike antigens in 33 participants whose sera were available from the fourth (2019) to the seventh year (2022). Antibodies against the MERS-CoV S1 antigen gradually decreased (Fig. 2A), as seen in Fig. 1A. In contrast, the antibody responses against the S2 antigen and full-spike ectodomain antigen (S) gradually increased in the sixth and seventh years (Fig. 2, B and C). In addition, there was a slight upward trend in the neutralizing activity of the sera throughout the study period (Fig. 2D). However, all these changes in antibody levels were marginal and not statistically significant. Nevertheless, gradual increases in antibody responses [anti-S2 and S immunoglobulin G (IgG)] and neutralizing activities were consistently observed in all patient groups, although the antibody responses were generally higher in patients who recovered from MERS pneumonia (G II and G III) than in those without pneumonia (G I) (Fig. 2, E to H). COVID-19 vaccination and infection history of the 33 patients revealed that majority of them (93.9%) were immunized with various combinations of commercial vaccines in the sixth and seventh years (table S2 and fig. S1). Moreover, nine participants (37.3%) experienced confirmed SARS-CoV-2 infection in the sixth or seventh year. On comparing mean neutralizing activities before and after exposure to the COVID-19 vaccine and/or SARS-CoV-2 infection, 17 participants showed an increase in nAb responses against MERS-CoV, with seven participants demonstrating >50% increase in neutralizing activity (table S2 and Fig. 2I). In contrast, the remaining 16 participants showed decreased neutralizing activity, indicating individual variations in antibody responses against MERS-CoV during the COVID-19 pandemic. On the basis of our clinical data, changes in neutralizing activity were not significantly associated with MERS severity, age, sex, or SARS-CoV-2 infection history, although male patients who recovered from severe MERS generally presented higher neutralizing activity (fig. S1). nAb responses against MERS-CoV significantly correlated with antibody levels against S1, S2, and S antigens (Fig. 2J). Thus, the gradual increase in neutralizing activity against MERS-CoV after exposure to the COVID-19 vaccine and/or SARS-CoV-2 infection may be attributed to emerging cross-reactive antibody responses against S2, which is elevated during COVID-19 (*3*), than those against S1, which gradually decreased during the surveillance period (Fig. 2K). Given that antibody response to the S1 antigen strongly correlated with the neutralizing activity, these antibodies may still play a substantial role in the neutralization of MERS-CoV (Fig. 2J).

**Fig. 3.**
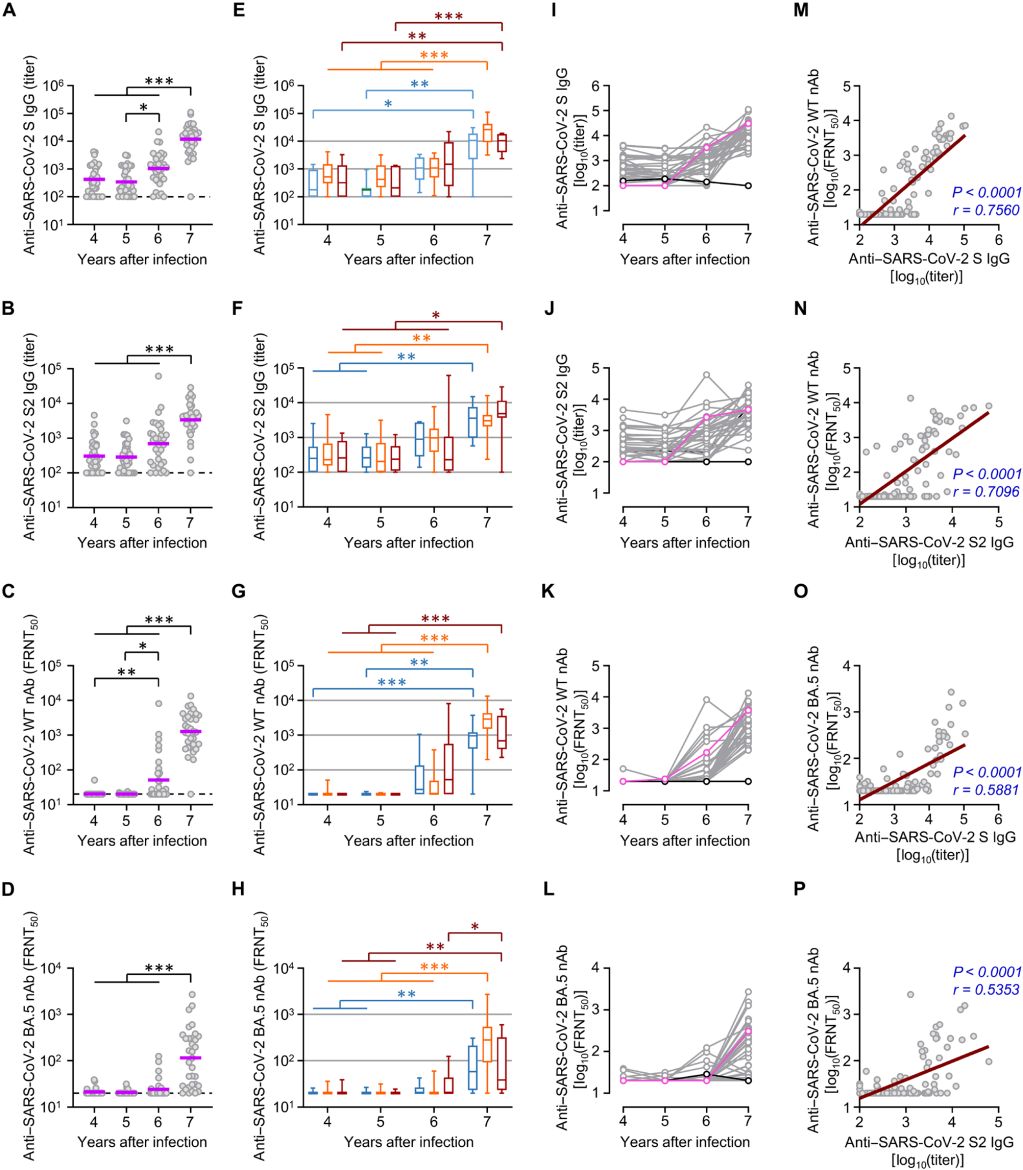
Kinetic changes in antibody responses against spike antigens and neutralizing activity against SARS-CoV-2. (**A** to **D**) Antibody response levels against indicated SARS-CoV-2 spike antigens and neutralizing activity 4 to 7 years after the Korean MERS outbreak. Purple line, geometric mean; nAb, neutralizing antibody; dashed line, limit of detection; *n* = 33 per year. (**E** to **H**) The antibody response levels against indicated SARS-CoV-2 spike antigens and neutralizing activity according to clinical MERS severity represented as box and whisker (minimum to maximum) plots, including the median value. *n* = 33 per year. (**I** to **L**) Kinetic changes in antibody levels against indicated spike antigens and neutralizing activity of individual participants (pink: unvaccinated and SARS-CoV-2–infected participant, black: unvaccinated and uninfected participant). (**M** to **P**) Correlation of neutralizing activity against SARS-CoV-2 wild type (WT) [(M) and (N)] or BA.5 variant [(O) and (P)] with the indicated antibody levels were assessed using linear regression (brown line) and Spearman’s rank test (*r* and *P* value in indigo). *P < 0.05; **P < 0.01; ***P < 0.001 using the Kruskal-Wallis test.

COVID-19 vaccination and SARS-CoV-2 infection induced specific antibody responses against SARS-CoV-2 spike antigens and neutralizing activity against SARS-CoV-2. Antibody responses specific to the SARS-CoV-2 spike S and S2 antigens were markedly elevated in the sixth and seventh years (Fig. 3, A and B). In addition, neutralizing activities against SARS-CoV-2 wild type and a recent variant, BA.5, were significantly enhanced in the seventh year (Fig. 3, C and D). Regardless of MERS severity, quantitative and kinetic trends in antibody responses were consistently observed in all participants (Fig. 3, E to H), except those who were unvaccinated and uninfected during the surveillance period (Fig. 3, I to L, black line). We also observed a significantly strong correlation between the neutralizing activity and specific IgG levels against SARS-CoV-2 S2 and S antigens (Fig. 3, M to P).

**Fig. 4.**
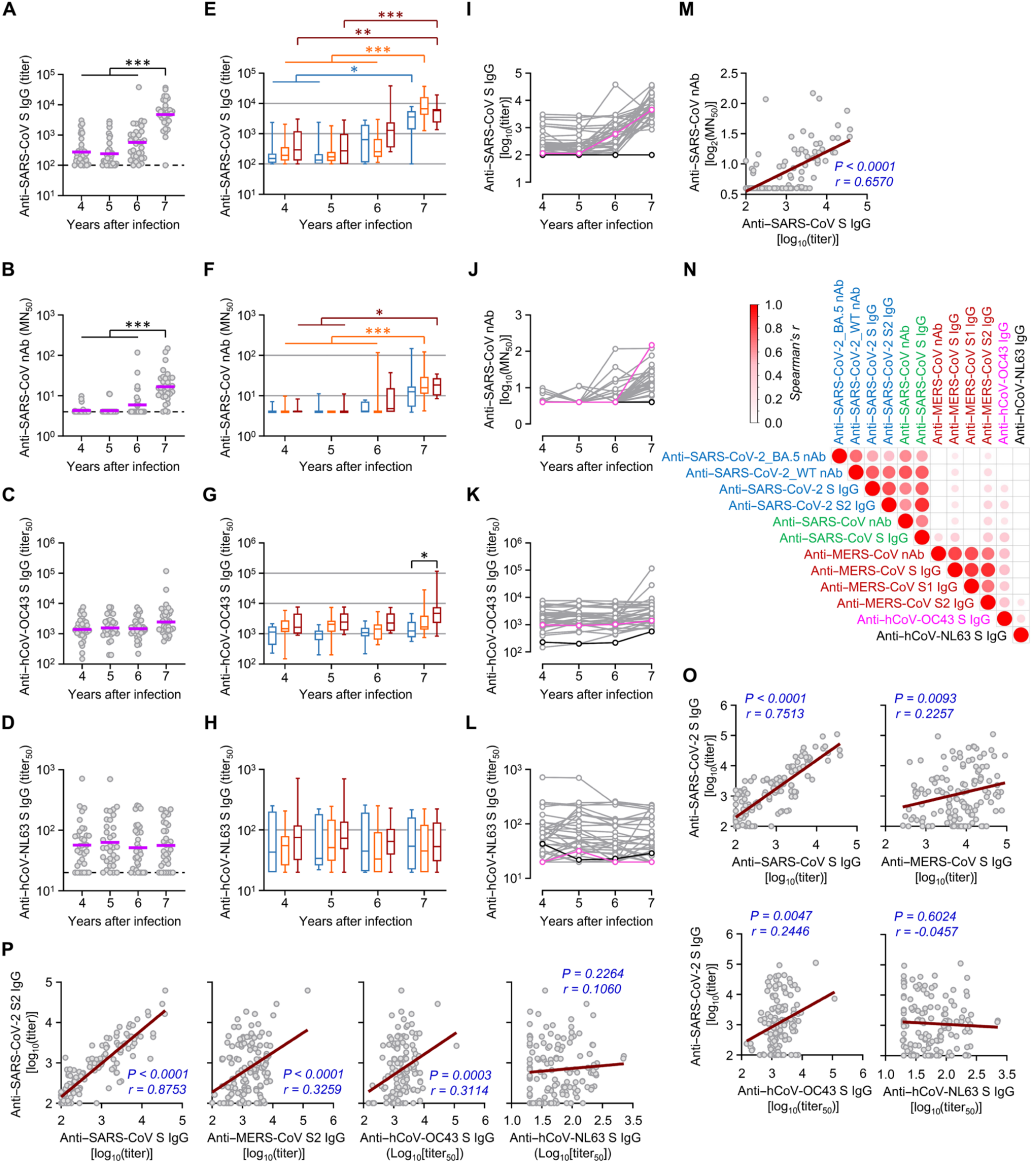
Kinetic changes in antibody responses against hCoV spike antigens and their correlation. (**A** to **D**) The antibody response levels against indicated hCoV spike antigens and neutralizing activity against SARS-CoV 4 to 7 years after the Korean MERS outbreak. Purple line, geometric mean; nAb, neutralizing antibody; dashed line, limit of detection; *n* = 33 per year. (**E** to **H**) The antibody response levels against indicated hCoV spike antigens and neutralizing activity according to clinical MERS severity represented as box and whisker (minimum to maximum) plots, including the median value. *n* = 33 per year. (**I** to **L**) Kinetic changes in individual antibody levels against indicated spike antigens and neutralizing activity (pink: unvaccinated and SARS-CoV-2–infected participant; black: unvaccinated and uninfected participant). (**M**) Correlation of neutralizing activity against SARS-CoV with anti–SARS-CoV S antibody levels was assessed using linear regression (brown line) and Spearman’s rank test (*r* and *P* value in indigo). (**N**) Correlation matrix of antibody levels against various hCoVs’ spike antigens and neutralizing activity. The circle sizes and color intensities are proportional to Spearman’s correlation coefficients. Only significant correlations (*P* < 0.05) are presented. *n* = 132. (**O** and **P**) Correlation of antibody levels against SARS-CoV-2 S antigen (O) or S2 antigen (P) with indicated hCoV’s S antigens is analyzed by Spearman’s rank test (*r* and *P* value in indigo). *P < 0.05; **P < 0.01; ***P < 0.001 using the Kruskal-Wallis test.

Furthermore, we assessed antibody responses against other hCoVs, including SARS-CoV, hCoV-OC43, and hCoV-NL63. Anti–SARS-CoV S IgG and neutralizing activity during the pandemic gradually increased, with significantly higher antibody levels in the seventh year than those in the fourth, fifth, and sixth years (Fig. 4, A and B). Regardless of MERS severity, quantitative and kinetic trends in antibody responses were consistently observed in all participants (Fig. 4, E and F), except one participant who was unvaccinated and uninfected during the surveillance period (Fig. 4, I and J). The antibody levels against the SARS-CoV S antigen also positively correlated with neutralizing activity against the virus (Fig. 4M). Considering no SARS-CoV exposure history, this increase in spike-specific antibodies strongly suggests the emergence of cross-reactive antibodies after COVID-19 vaccination and/or SARS-CoV-2 infection. Moreover, we detected a marginal increase in specific antibodies against hCoV-OC43 in the seventh year (Fig. 4C), where G III patients presented significantly higher levels (mean titer_50_: 4952.8) of anti–hCoV-OC43 S IgG than those (mean titer_50_: 1393.2) in G I participants (Fig. 4G). Among the 33 participants, 25 (75.8%) presented with increased specific antibody responses in the seventh year compared to those in the sixth year, whereas eight participants (24.2%) showed a marginal reduction in antibody responses against hCoV-OC43 during the pandemic (Fig. 4K). Two participants presented markedly increased specific IgG responses against the phylogenetically distant endemic hCoV-OC43 by 17- and 65-fold in the seventh year compared to those in the sixth year (Fig. 4K), suggesting that some MERS-recovered patients had potent cross-reactive antibodies produced by memory B cells, or they may have been infected with hCoV-OC43 during the pandemic. In contrast, antibody responses against hCoV-NL63 spike antigen were not significantly affected during the pandemic (Fig. 4, D, H, and L). Thus, a specific increase in broadly cross-reactive antibodies against human βCoVs was induced in some MERS-recovered patients by COVID-19 vaccination and/or SARS-CoV-2 infection during the pandemic; however, these responses might be barely reactive to other human αCoVs. In addition, we failed to detect significant changes in specific IgG levels against other respiratory viruses such as human influenza A virus and rhinovirus during the pandemic (fig. S2).

### Positive correlation of antibody levels against human βCoVs in MERS-recovered patients

**Fig. 5.**
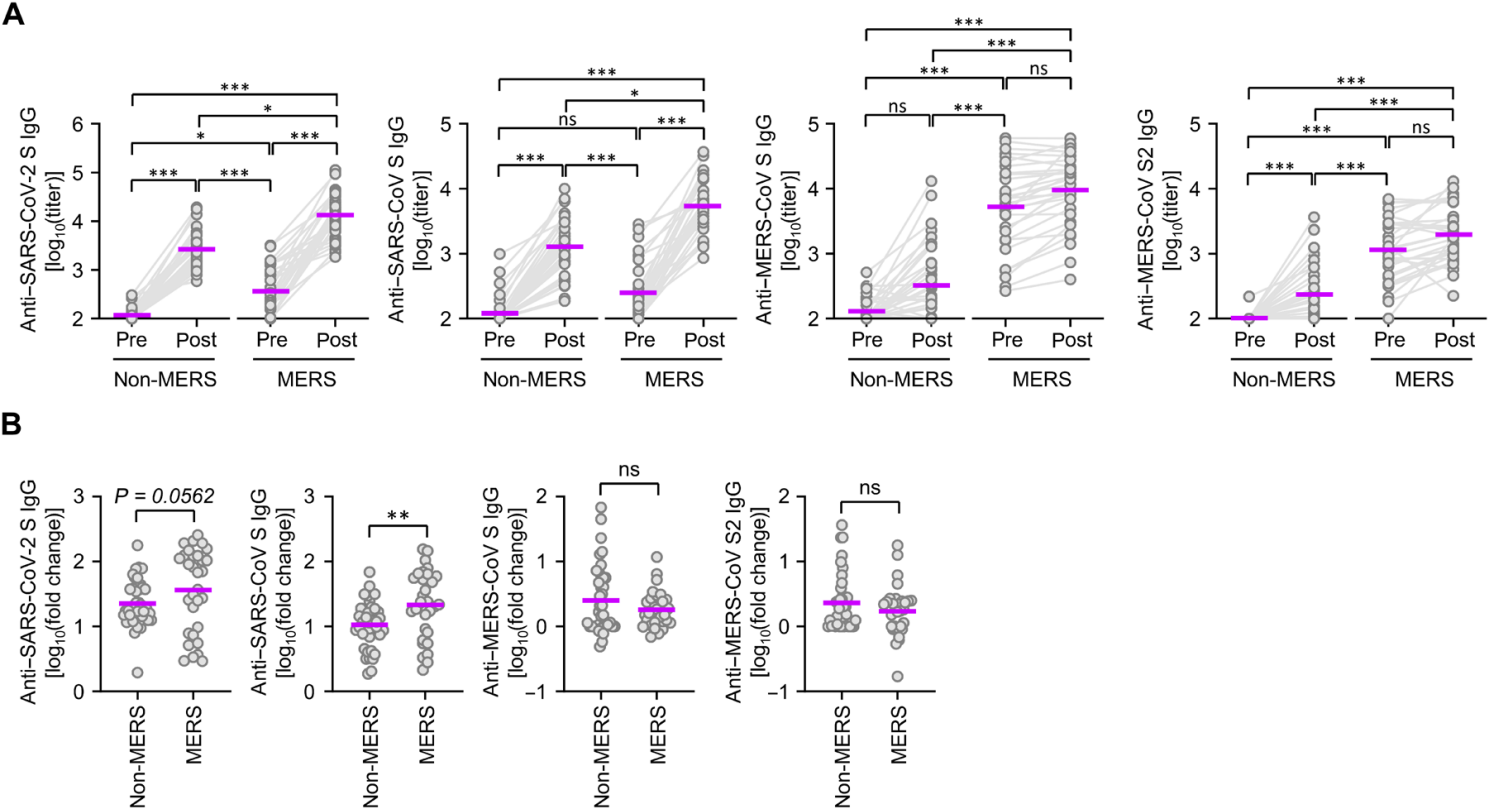
Comparison of antibody responses against zoonotic CoV’s spike antigens between non-MERS and MERS-recovered group after COVID-19 vaccination. (**A**) Comparison of antibody responses to indicated CoV spike antigens in the non-MERS control group (*n* = 36) and the MERS-recovered cohort (*n* = 31) before (pre) and after (post) COVID-19 vaccination. Purple line, geometric mean. *P < 0.05; **P < 0.01; ***P < 0.001 by Kruskal-Wallis test. ns, not significant. (**B**) Comparison of fold increases in antibody responses to indicated CoV spike antigens between the non-MERS control group and the MERS-recovered cohort before and after COVID-19 vaccination. *P* value calculated using the Mann-Whitney test.

The above findings indicated potential correlations among specific antibody responses in our MERS-recovered cohort, which was confirmed using pairwise correlation analysis (Fig. 4, N to P). Antibody responses against hCoV-OC43, an endemic embecovirus, significantly correlated with those against sarbecoviruses (SARS-CoV-2 and SARS-CoV) and merbecovirus (MERS-CoV). Antibody responses against hCoV-NL63 exhibited a positive correlation solely with antibodies against MERS-CoV S2 and hCoV-OC43 but barely correlated with antibodies against other βCoV’s spike antigens (Fig. 4, N to P). These results are consistent with the amino acid sequence conservation rate of spike antigens (S2 > S > S1) among hCoVs (fig. S3). We also confirmed the significant induction of antibodies against the spike antigens of SARS-CoV-2 and SARS-CoV after COVID-19 vaccination in healthy controls unexposed to MERS-CoV (Fig. 5A). In addition, the control group exhibited a significant rise in the antibody response to the S2 antigen of MERS-CoV. However, before vaccination, the control group’s baseline antibody levels against the S antigens of SARS-CoV-2 and MERS-CoV were significantly lower than those of MERS-recovered patients who had not been vaccinated against COVID-19 (fifth year). MERS-recovered patients in their seventh year also showed notably elevated antibody levels for SARS-CoV-2 S, SARS-CoV S, and MERS-CoV S/S2 antigens compared to the control group after COVID-19 vaccination. For the MERS-recovered group in the seventh year, postvaccination antibody levels for S antigens of SARS-CoV-2 (mean titer ± SD: 21,953 ± 24,768), SARS-CoV (8266 ± 8653), and MERS-CoV (16,762 ± 15,817) were significantly higher than those in the non-MERS controls after vaccination (anti–SARS-CoV-2 S: 3873 ± 4271, anti–SARS-CoV S: 1975 ± 2063, and anti–MERS-CoV S: 1012 ± 2396). Moreover, the antibody fold increase specific to the S antigens of SARS-CoV-2 and SARS-CoV after COVID-19 vaccination was also substantially higher in the MERS-recovered group (fifth versus seventh year) relative to the non-MERS control group (Fig. 5B). Thus, prior exposure to MERS-CoV spike antigens might further contribute to the antibody responses against SARS-CoV-2 and SARS-CoV spike antigens after COVID-19 vaccination. The antibody fold increase for S and S2 antigens of MERS-CoV was not significantly different between the non-MERS control and MERS-recovered groups. This may be due to the preexisting higher antibody levels in MERS-recovered subjects.

### Landscaping linear epitopes in hCoVs’ spikes recognized by cross-reacting antibodies in MERS-recovered patients

**Fig. 6.**
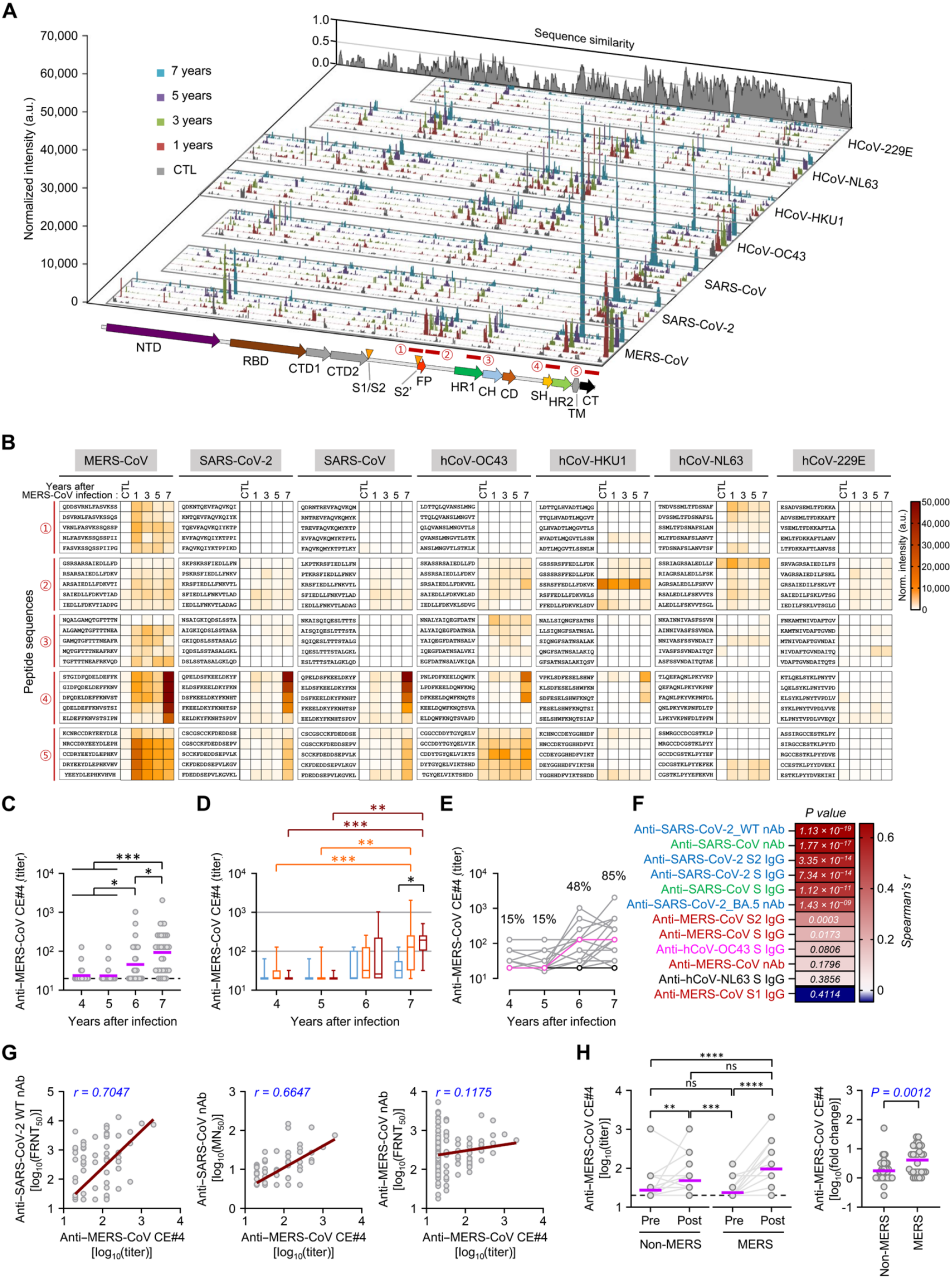
Kinetic changes in antibody responses against hCoVs’ linear spike epitopes and their correlation with antibody levels against hCoV’s spikes. (**A**) Landscape of antibody responses against overlapping 15-mer peptides derived from various hCoVs’ spikes. The width of the antigenic peak for each spike protein was adjusted approximately, considering sequence similarities and lengths. Broadly reactive representative peptide region (① to ⑤) within S2 antigens was selected. CTL, negative control pooled sera. (**B**) Peptide sequences in the five regions and kinetic responses against them. (**C**) The antibody response levels against CE#4. Purple line: geometric mean; dashed line: limit of detection; *n* = 33 per year. (**D**) Antibody response levels against CE#4, according to clinical MERS severity. (**E**) Kinetic changes in individual serum antibody levels against CE#4 (pink: unvaccinated and SARS-CoV-2–infected participant; black: unvaccinated and uninfected participant). Seropositive rate of each year is also presented. (**F**) Correlation of anti-CE#4 antibody with antibody levels against various hCoVs’ spike antigens assessed using Spearman’s rank test. *n* = 132. (**G**) Correlation of anti-CE#4 antibody with indicated neutralizing activity assessed using linear regression (brown line) and Spearman’s rank test. (**H**) Change in serum anti-CE#4 antibody levels before and after COVID-19 vaccination. *P < 0.05; **P < 0.01; ***P < 0.001 by Kruskal-Wallis test or the Mann-Whitney test. a.u., arbitrary units.

Furthermore, the antibody responses cross-reacting with hCoVs were characterized using a microarray system. Sera collected from MERS-recovered patients in the first, third, fifth, or seventh year after MERS-CoV infection were pooled and screened for reactive linear epitopes. Pooled sera from healthy controls, unexposed to MERS-CoV or SARS-CoV-2 antigens, were used as negative controls. Antibody reactivities, presented by normalized intensity values, were arranged on sequence alignments of hCoVs’ spike domains. This approach allowed us to landscape linear epitopes reacted by serum antibodies and quantify the kinetic responses of the reactive antibodies (fig. S4A). A general temporal increase in the overall binding intensities against the spike antigens derived from the seven hCoVs was observed (fig. S4B). Among the peptide epitopes, we identified five immunodominant epitopes within S2 antigens, which were more conserved than the S1 subunits and present broad reactivity across hCoVs (Fig. 6). These include a region [common epitope #2 (CE#2)] encompassing the fusion peptide, CE#4 containing a conserved stem-helix region, and the cytoplasmic tail (CE#5) of human CoVs. CE#2 and CE#4 are highly conserved among diverse CoVs and exhibit the broadest neutralizing spectrum by preventing S2 cleavage and the fusion of viral and cellular membranes, respectively (*3*). In particular, the binding capacity of antibodies against CE#4 increased for all five human βCoVs in the pooled sera collected in the seventh year (Fig. 6, A and B). This strongly suggests an active boosting of the antibody repertoire against the conserved stem helix region of the spike antigen by COVID-19 vaccination and/or SARS-CoV-2 infection. We confirmed that antibody responses against MERS-CoV CE#4 were elevated in all MERS-recovered patients, except one participant unvaccinated and uninfected with SARS-CoV-2, during the pandemic period (sixth or seventh year) (Fig. 6, C to E). Notably, this antibody response was significantly higher in G III than in G I in the seventh year (Fig. 6D). A pairwise correlation analysis involving antibodies against various spike antigens showed the most robust positive correlation between antibody levels against MERS-CoV CE#4 and neutralizing activity against SARS-CoV-2 and SARS-CoV (Fig. 6, F and G). This was also observed with antibody levels against S antigens (S or S2) of SARS-CoV-2, SARS-CoV, and MERS-CoV. However, there was moderate to no significant correlation with antibodies against hCoV-OC43 S, MERS-CoV S1, and the neutralizing activity against MERS-CoV. The antibody levels against MERS-CoV CE#4 were also significantly increased in the non-MERS control group after COVID-19 vaccination (Fig. 6H). However, the specific antibody levels were elevated in only 56.8% (21 of 37), unchanged in 37.8% (14 of 37), and decreased in 5.4% (2 of 37) of cases after vaccination, compared to those before vaccination. This antibody response increased in 84.8% (28 of 31) of MERS-recovered group after vaccination. In addition, the fold increase in antibody levels specific to CE#4 of MERS-CoV’s spike antigen was significantly higher in the MERS-recovered group compared to the non-MERS control group (Fig. 6H, right). Again, prior exposure to MERS-CoV spike antigens may better amplify the antibody response to the conserved CE#4 (stem helix epitope) in MERS-recovered subjects after COVID-19 vaccination, more so than in the non-MERS group.

Notably, antibodies against βCoV’s CE#5 (cytoplasmic tail) were moderately induced in the first year after the MERS outbreak and sustained up to the seventh year, although variations were observed depending on the βCoV species (Fig. 6B and fig. S4C). The antibody response to CE#5 appears to be specific to the viral species. An enhanced response to CE#5 of MERS-CoV was detected in the first year, while responses to those of SARS-CoV-2 and SARS-CoV increased during the pandemic.

### Kinetic changes in memory “T cell” responses against MERS-CoV during the COVID-19 pandemic

**Fig. 7.**
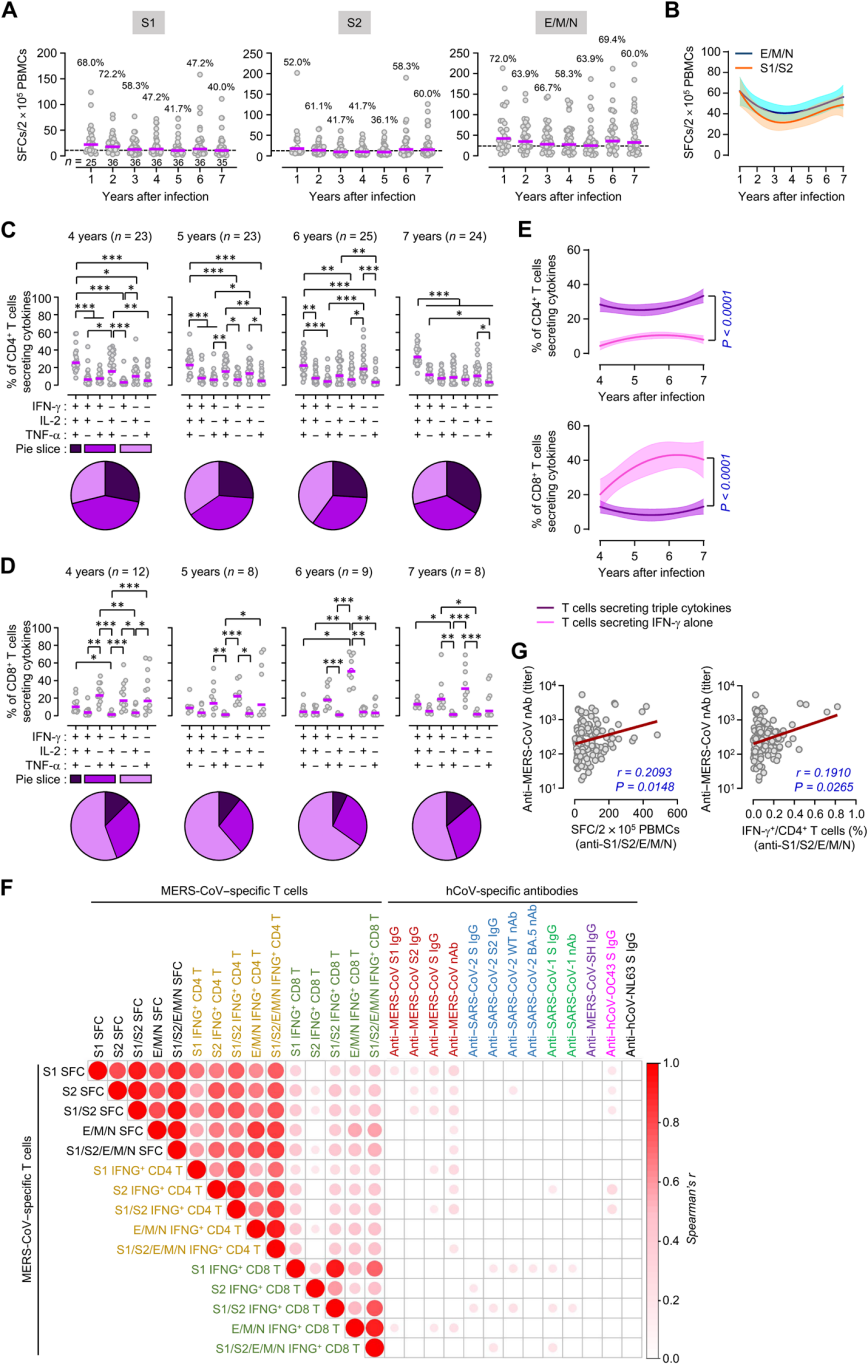
Kinetic changes in MERS-CoV–specific T cell responses and their correlation with antibody responses against various hCoVs’ spike antigens and neutralizing activity. (**A**) Ex vivo IFN-γ ELISpot responses to MERS-CoV structural proteins. The annual positivity rates of memory T cell responses (over mean + 2 × SD of healthy controls, dashed line). Purple line, geometric mean; SFC, spot-forming cell; PBMC, peripheral blood mononuclear cell. (**B**) Mean memory T cell response kinetics after nonlinear regression analysis (solid line) with 95% CI (shaded color). (**C** and **D**) Cytokine-production profiles of the MERS-CoV–specific CD4^+^ T cells (C) and CD8^+^ T cells (D) in the MERS-recovered cohort. All possible combinations of IFN-γ, IL-2, and TNF-α are shown on the *x* axis. The response at each year is grouped according to the number of functions and data summarized using pie charts. Each slice of the pie represents the fraction of the total response that consists of T cells positive for a given number of functions. Purple line, geometric mean. (**E**) Kinetic changes in the mean value of polyfunctional and IFN-γ^+^ memory T cell responses after nonlinear regression analysis (solid line) with 95% CI (shaded color). (**F**) Correlation matrix of MERS-CoV–specific memory T cells with antibody levels against various hCoVs’ spike antigens and neutralizing activity. The circle sizes and color intensities are proportional to Spearman’s correlation coefficients. Only significant correlations (*P* < 0.05) are presented. (**G**) Correlation of neutralizing acitivity against MERS-CoV with memory T cells specific to MERS-CoV’s structural peptide pools (S1/S2/E/M/N) assessed using linear regression (brown line) and Spearman’s rank test. *n* = 132.

Stimulation of peripheral blood mononuclear cells (PBMCs) with synthetic MERS-CoV peptides reveals a gradual decrease in interferon-γ (IFN-γ)–producing T cell levels in most participants, yielding a 64% overall positivity rate of memory T cell responses in the fifth year after infection (*15*). Here, the mean frequency of T lymphocytes increased in participants during the COVID-19 pandemic (Fig. 7A). The positivity rates of memory T cell responses specific to any of the viral antigens (S1, S2, E, M, and N) were 80.6 and 65.7% at the sixth and seventh year, respectively. Increase in the specific memory T cells were detected in a subset of participants [>1.5-fold increase in 44.4% (16 of 36)] during the pandemic. However, these annual changes did not reach statistical significance. The mean specific memory T cell frequencies were moderately elevated at the sixth and seventh year [mean ± SD, 103 ± 95 spot-forming cells (SFCs)/2 × 10^5^ PBMCs] when compared to those at the fourth and fifth year (74 ± 62 SFCs/2 × 10^5^ PBMCs) (Fig. 6B). This kinetic pattern was similar among all the severity groups (fig. S5A). The moderate increase may be attributed to cross-priming by COVID-19 vaccination and/or SARS-CoV-2 infection, as observed in the antibody responses. Simultaneous increases in the frequency of SARS-CoV-2– and MERS-CoV–reactive T cells observed in several MERS-recovered patients who were vaccinated and infected with SARS-CoV-2 during the sixth and seventh years indirectly supported this possibility (fig. S6, A and F). In addition, an increase in the positivity rate of IFN-γ^+^ T cells was observed in cells stimulated with S2 peptides (36.1, 58.3, and 60.0% at the fifth, sixth, and seventh year, respectively) (Fig. 7A, middle). Because a subset of participants displayed an increase in specific T cell responses against E/M/N (fig. S6A), which were not included in COVID-19 vaccines, these responses might have been induced by cross-active T cells after exposure to SARS-CoV-2 and/or other βCoVs during the pandemic. In general, these responses were higher than those of the specific T cells responding to the spike protein (S1 or S2) (Fig. 7, A and B). However, none of the annual changes in T cell responses were statistically significant, and they occurred at moderate levels.

Furthermore, we determined which subset of T lymphocytes participated in persistent memory T cell responses by performing intracellular cytokine staining after the stimulation of PBMCs with viral peptides. MERS-CoV–reactive IFN-γ–secreting T cells generally persisted in the participants up to the seventh year, with a gradual rise in some participants at the sixth and seventh years (fig. S6, B to D), similar to that observed for antibody responses. The frequency of virus-reactive cells in CD4^+^ T and CD8^+^ T lymphocytes also tended to constantly persist and even increased in some patients during the pandemic, driving a gradual rise in the mean frequency of the memory T cell subsets (fig. S6D). A rising trend (>1.5-fold increase) during the pandemic was observed in both CD4^+^ (38.9% of participants) and CD8^+^ T cells (52.8% of participants) when compared to those in the fourth and fifth years, whereas only two (5.6%) and five (13.9%) participants presented >50% reduction in antigen-specific CD4^+^ and CD8^+^ T cells. In addition, the CD4^+^ T cell frequency positively correlated with that of CD8^+^ T cells observed in the fourth to seventh years (fig. S6E). Both CD4^+^ and CD8^+^ T cells generally responded better to E/M/N proteins than to S protein antigens during the follow-up period (fig. S6D), as reported previously (*15*). Notably, the frequency of SARS-CoV-2 antigen–specific CD4^+^ and CD8^+^ T cells simultaneously increased in participants during the sixth and seventh years (fig. S6G), suggesting that the increase in MERS-CoV–specific T cell responses might have been induced by cross-reactive T cells after vaccination and/or SARS-CoV-2 infection. Nonetheless, there was no significant difference in the levels of MERS-CoV–specific memory CD4^+^ and CD8^+^ T cells among the three different severity groups and few correlations with age, sex, and SARS-CoV-2 infection history in our cohort [fig. S5 (B and C) and S7A].

We also examined the polyfunctionality of memory T cells that secrete multiple cytokines, such as IFN-γ, tumor necrosis factor–α (TNF-α), and interleukin-2 (IL-2), upon antigenic (S1/S2/E/M/N) stimulation. MERS-CoV-reactive CD4^+^ T cells showed a similar distribution in single-, double-, and triple-cytokine–producing cells, whereas single-cytokine–producing cells were dominant in memory CD8^+^ T cells (Fig. 7, C and D). Notably, IFN-γ^+^ CD8^+^ T cells were highly up-regulated in the sixth and seventh years (Fig. 7E). Predominant multifunctional CD4^+^ T cells together with enhanced IFN-γ^+^ CD8^+^ T cells specific to SARS-CoV-2 antigens after viral infection have been reported previously (*17*, *18*). Therefore, potential cross-reactive T cell responses induced by COVID-19 vaccination and/or SARS-CoV-2 infection may drive antigen-specific memory T cell responses against MERS-CoV (*19*).

### Correlations between memory T cell and antibody responses

We further assessed the correlation between antigen-specific antibodies and T cell responses observed in MERS-recovered patients from the fourth to seventh years. Using pairwise comparisons, MERS-CoV–specific T cells measured using enzyme-linked immunosorbent spot (ELISpot) analysis and flow cytometry after stimulation with mixed peptide pools derived from the S/E/M/N antigens extensively correlated with each other (Fig. 7F). In particular, CD4^+^ T cells significantly correlated with the nAb titers against MERS-CoV (Fig. 7, F and G). Antibody responses against MERS-CoV S1, SARS-CoV-2, and SARS-CoV barely correlated with T cell responses. However, we observed a broader and more significant correlation between MERS-CoV S antigen-specific T cells and antibody levels against MERS-CoV S/S2 and hCoV-OC43 (Fig. 7F and fig. S7B). Thus, broadly cross-reacting spike-specific T cell responses, especially against the S2 subunit generated by COVID-19 vaccination, may induce broadly cross-reacting antibodies against β-CoVs as well as neutralizing activities, but not against the highly variable S1 subunit. Furthermore, IFN-γ–secreting CD4^+^ T cells specific to MERS-CoV antigens significantly correlated (*P* = 0.0265) with nAb levels against MERS-CoV, whereas those of CD8^+^ T cells, except cells stimulated with E/M/N antigens, failed to do so (fig. S7, C and D). Therefore, memory B cells with broadly cross-reactive neutralizing potential against βCoVs observed in MERS-recovered patients might be boosted by elevated CD4^+^ T cells induced by COVID-19 vaccination and/or SARS-CoV-2 infection.

## DISCUSSION

Information on the characteristics, longevity, and cross-reactivity of antibodies and memory T cell responses after acute CoV infection is important for developing effective control strategies. Our long-term follow-up study with a well-defined cohort of MERS-recovered patients revealed the following characteristics of humoral and cellular memory responses.

First, antibody responses and neutralizing activity against MERS-CoV peaked 1 to 2 years after infection and gradually declined thereafter (Fig. 1). The estimated half-lives of anti–MERS-CoV S1 IgG and nAbs in seropositive participants during 5 years after the MERS outbreak are 61 and 20 months, respectively (*16*). Virus-specific antibody responses and memory T lymphocytes were detected in 17.1% (anti–MERS-CoV S1 IgG) and 65.7% (IFN-γ^+^ cells) of the survivors 7 years after infection, indicating a relatively longer persistence of memory T cells than that of antibody responses. Memory T cells predominated in the CD4^+^ T lymphocyte compartment, although CD8^+^ T cells were also found in some participants (fig. S6). Specific IgG antibodies to SARS-CoV have been majorly undetectable in patients with SARS (8.7% positivity) 6 years after infection, along with the absence of SARS-CoV–specific memory B cell responses (*20*). However, memory T cell responses to a pool of SARS-CoV S peptides have been detected in 60.9% of patients 6 years after recovery (*20*) and long-lasting memory T cells reactive to SARS-CoV N protein have been detected 17 years after the 2003 SARS outbreak (*4*). Thus, memory T cell immunity lasting for >7 years has been observed in at least 50% of MERS-CoV–infected participants, which is similar to that acquired from SARS-CoV infection. Moreover, MERS-specific cellular memory responses could last for up to 6.9 years, although this study was performed with a small number of MERS survivors at single time points (*21*). Antibody responses and neutralizing activity against viral antigens during SARS-CoV-2 infection generally peak in the first month after infection, gradually decline, and stabilize 4 to 6 months after infection (*22*). The estimated half-life of the SARS-CoV-2 nAb is >200 days, and neutralizing activity is detectable in approximately 80 to 90% of SARS-CoV-2–infected individuals 12 months after infection (*23*, *24*). The estimated half-lives of SARS-CoV-2–specific memory CD4^+^ and CD8^+^ T cell kinetics vary widely from 100 to 400 days and 100 to 200 days, respectively, and long-lasting memory T cells exist against SARS-CoV-2 (*22*). Although long-term follow-up studies on the antibody and memory T cell responses against SARS-CoV-2 are required to confirm the longevity of adaptive immunity and for comparative analysis with MERS-CoV and SARS-CoV infections, active vaccine campaigns and the continuous emergence of SARS-CoV-2 variants may hamper this approach.

Second, the antibody levels among patients in G II and G III were consistently higher than those in G I throughout the surveillance period. Moreover, the initial antibody responses in patients with severe MERS (G III) were delayed compared to those in patients with moderate pneumonia (G II) (*25*), whereas more rapid and robust antibody responses specific to SARS-CoV-2 antigens are consistently observed in patients with severe COVID-19, potentially due to extrafollicular B cell activation (*26*–*28*). In addition, the longevity of specific antibodies in patients with COVID-19 (less than 1 year) is considerably shorter than that in patients with MERS (approximately 5 years) (*16*, *29*). Thus, the differential mechanisms modulating initial antibody responses, their role in disease progression, and the regulatory networks controlling the longevity of memory responses among various zoonotic CoV infections, depending on their virulence in humans, need to be further characterized (*30*). Nonetheless, prolonged antibody responses and neutralizing activity in nonhospitalized patients who recovered from more severe MERS are observed for up to 6 years after infection (*21*). Although the frequency of memory T lymphocytes is high in severely or moderately ill patients than that in mildly ill patients during early recovery from infection (*14*), this difference gradually disappeared (fig. S5).

Third, antibody and memory T cell responses against MERS-CoV were boosted by other βCoV infections and/or vaccination, such as SARS-CoV-2, only in some MERS-recovered patients. The degree of antibody and memory T cell resurgence against MERS-CoV may be independent of initial MERS severity. Memory T lymphocytes, particularly CD4^+^ T cells, responded better to E/M/N proteins than to the S protein of MERS-CoV (fig. S6). In addition, the polyfunctionality of these memory T lymphocytes did not significantly change during the follow-up period but was slightly enhanced during the pandemic, potentially due to cross-priming through SARS-CoV-2 vaccination and/or infection, similar to the boost in preexisting cross-reactive memory T cells in SARS-CoV-1 survivors after COVID-19 vaccination (*31*). In addition, COVID-19 vaccination or SARS-CoV-2 infection can induce T cell proliferation with cross-reactivity to MERS-CoV in individuals previously uninfected with MERS-CoV (*32*). The simultaneous increase in MERS-CoV– and SARS-CoV-2–reactive T cells in some MERS survivors infected with SARS-CoV-2 and/or vaccinated also suggests cross-reactivity (fig. S6). Therefore, the increase in MERS-CoV–reactive memory T cell numbers observed in only some patients during the pandemic might be caused by exposure to SARS-CoV-2 antigens; however, this needs further investigation. The positive correlation of the memory T cell responses, particularly CD4^+^ T cells, with the MERS-CoV antibodies suggested that the boosted antibody responses might be supported by antigen-specific memory T cells (Fig. 7F) and cross-reactive memory B cell responses against conserved epitopes such as S2, as revealed by correlation analysis using a dataset of antibody responses against various hCoVs (Fig. 4). Unbiased clustering based on all antibody response datasets revealed two groups in our MERS cohort, one with high and sustained anti–MERS-CoV antibodies and the other with relatively low anti–MERS-CoV antibodies (fig. S1). The first group was male-dominated (82.4%, 14 of 17) and recovered from more severe MERS (G II: 58.8%, G III: 41.2%), whereas the second group was female-dominated (62.5%, 10 of 16) and generally exhibited milder MERS (G I: 43.8%, G II: 31.3%, and G III: 25.0%). Although the first group presented more enhanced antibody responses against hCoV-OC43, which correlated better with MERS-CoV antibodies than with SARS-CoV-2 or SARS-CoV antibodies (Fig. 4N), both groups showed similar degrees of elevation in anti–SARS-CoV-2 and SARS-CoV antibodies, indicating that the COVID-19 vaccination equally cross-boosted anti–SARS-CoV antibodies, regardless of previous anti–MERS-CoV antibody levels. However, we observed a remarkable increase in cross-reactive antibody responses against the conserved CE#4 linear epitope in MERS survivors, especially in G III group recovered from severe MERS (Fig. 6D). The antibody levels at the seventh year were significantly higher (*P* = 0.0068) in the first group [mean log_2_(titer) ± SD, 7.2 ± 2.1] than those in the second group (5.5 ± 1.6) (fig. S1). In addition, the fold increase in antibody levels against the SARS-CoV S antigen and the MERS-CoV CE#4 epitope in MERS survivors was notably higher than that observed in the non-MERS group following COVID-19 vaccination (Figs. 5B and 6H). Moreover, prior exposure to MERS-CoV spike antigens contributed to significantly higher antibody responses against MERS-CoV, SARS-CoV-2, and SARS-CoV spike antigens when compared to non-MERS group after COVID-19 vaccination (Fig. 5A). Previous studies also reported induction of cross-reactive antibody responses in MERS-recovered patients during the COVID-19 pandemic, although they examined a smaller cohort (*33*, *34*). Thus, stronger and sustained anti–MERS-CoV antibody responses and memory B cells after recovery may support enhanced cross-reactivity of antibodies against the conserved stem helix epitopes of various human βCoVs, as well as those against βCoVs’ spike antigens, after COVID-19 vaccination and/or SARS-CoV-2 infection. Broadly neutralizing monoclonal antibodies against this epitope protect against all three human βCoVs in an in vivo infection model (*35*). Nine patients with confirmed COVID-19 in our cohort presented with only mild respiratory symptoms without pneumonia. Antibodies specific to the stem helix of various human βCoVs are less frequent, implying its low immunogenicity, in individuals previously infected with SARS-CoV-2 (7 to 44%) or completely vaccinated against COVID-19 (22 to 76%) (*36*). Notably, specific antibodies to MERS-CoV CE#4 were elevated in 56.8% vaccinated participants unexposed to MERS-CoV, whereas the antibody response increased in 84.8% of the MERS-recovered group after vaccination (Fig. 6H). Thus, if there are preexisting memory responses, even those established up to 7 years prior against other human βCoVs including MERS-CoV, plasma antibody responses to the stem helix are robustly triggered by SARS-CoV-2 infection or vaccination. Nonetheless, antibodies specific to MERS-CoV CE#4 demonstrate a significant correlation with neutralizing activity against SARS-CoV-2 and SARS-CoV, but only a marginal correlation with neutralizing antibodies against MERS-CoV (Fig. 6, F and G). Considering that antibody levels against the MERS-CoV S1 antigen, which includes the receptor-binding domain, have remained relatively high in our MERS cohort even during the pandemic (Fig. 1) and exhibit a strong correlation with neutralizing activity against MERS-CoV (Figs. 2J and 4N), the contribution of antibodies specific to the S1 antigen may be more substantial than those targeting MERS-CoV CE#4, which are induced by COVID-19 vaccination and/or SARS-CoV-2 infection.

Peptide arrays also identified the epitope CE#5 (Fig. 5) as a potential cytoplasmic epitope representing immunogenic and conserved epitopes of human βCoVs, which can be boosted by repeated infection and/or vaccination with heterologous spike antigens. However, the immunological role of antibodies against the intracellular domain of hCoV needs further investigation. We also observed relative increase in antibody levels against the highly conserved epitope (CE#2) containing the S2′ fusion peptide region of all CoV genera, in the first year after the MERS outbreak (Fig. 6, A and B) (*3*). Although these antibodies may provide excellent broad-neutralizing activities against all seven hCoVs (*37*), antibodies against this epitope in humans were barely boosted upon COVID-19 vaccination and/or SARS-CoV-2 infection, suggesting a relatively weaker immunogenicity than those against CE#4 and CE#5 (fig. S4C). Nonetheless, we noted a general increase in antibody levels against the spike peptide pools following MERS-CoV infection (from baseline to the first year) and during the pandemic (from the fifth to the seventh year) despite wide variations depending on the βCoV species.

The efficacy of vaccines against SARS-CoV-2 through nAb generation gradually weakens with the appearance of variant viruses and the rapid disappearance of nAbs in humans (*38*). Virus-reactive T cells are also generated upon vaccination and are critical in preventing disease development and progression to severe infection (*39*). Therefore, the active induction of long-lasting antibodies with broad neutralizing activity and memory T cells against various hCoVs by targeting conserved epitopes should be optimized to develop an effective pan-CoV universal vaccine. The inclusion of immunogenic epitopes within spike antigens, which can be easily boosted by repeatedly vaccinating the general human population and potently induce broad neutralizing activity against various hCoVs, could be extremely beneficial. In addition, the generation of long-lasting memory T cells, particularly CD4^+^ T cells, is also a promising strategy for developing effective pan-CoV vaccines. Recent reports suggest that the sustained presence and functional role of spike-specific CD4^+^ follicular helper T cells following SARS-CoV-2 infection and vaccination may be crucial for the maintenance of antibodies and the recall response, potentially offering long-term protection (*40*, *41*). Considering the hCoV species–dependent heterogeneity of immunogenic T cell epitopes within structural proteins (*39*, *42*), the identification and incorporation of cross-reactive T cell epitopes into vaccine formulations should be considered.

The limitations of this study include a relatively small sample size across all time points. Together with the heterogeneity of the measures depending on individual participants, it was difficult to draw statistically significant conclusions in several assays. The characterization and functionality of memory B cells producing broad nAbs against conserved epitopes in the viral spikes of various hCoVs need to be pursued in future studies. In addition, further research is required to characterize the continuously strong or belatedly increased T cell responses to MERS-CoV peptide pools observed in some MERS survivors, as this could considerably aid in interpreting memory T cell responses. Nonetheless, current information on the longevity and characteristics of antibody and memory T cell responses obtained from MERS-CoV infection in humans is valuable for developing effective infection control strategies. Moreover, this cohort also enabled us to investigate the impact of the COVID-19 pandemic on memory responses to the 2015 MERS-CoV infection. These study findings may clarify the development of pan-CoV vaccines and therapeutic antibodies, which can protect against emerging SARS-CoV-2 variants, MERS-CoV, and future novel CoVs.

## MATERIALS AND METHODS

### Study design and participants

Our Korean MERS cohort study initially recruited 70 MERS-recovered patients. Their baseline characteristics are presented in table S1 (*13*, *16*). All participants tested positive for MERS-CoV infection using a real-time reverse transcriptase polymerase chain reaction assay targeting the *upE* and *orf1a* viral sequences at a diagnostic laboratory in the Korean Center for Disease Control. During the 7-year study period, 35 participants were lost to follow-up, with 35 participants remaining in the seventh year (2022) after infection in 2015. Serum and PBMCs were collected from the participants at 12-month intervals after symptom onset. Data for the first 3 and 5 years have been assessed in our previous studies (*13*, *16*) and are included in this study. We used sera collected from 36 healthy controls who never exposed to MERS-CoV, SARS-CoV-2, and COVID-19 vaccine. These control sera were used for baseline cutoff antibody titer against βCoVs. The COVID-19 vaccine control group, consisting of 39 individuals who had not been exposed to MERS-CoV and had received COVID-19 vaccines between 2021 and 2022, provided serum samples. The study was approved by the Institutional Review Boards of Chungnam National University Hospital (CNUH2017-12-004), National Medical Center (H-1510-059-007), Seoul National University Hospital (1509-103-705 and 1511-117-723), Seoul National University Boramae Medical Center (26-2016-8), Seoul Medical Center (Seoul2015-12-102), and Dankook University Hospital (DKUH2016-02-014). This study was conducted in accordance with the ethical standards of the 1964 Declaration of Helsinki and all subsequent revisions. All study participants provided written informed consent. All other materials and methods are available in the Supplementary Materials.
