# Supplementary material for: Rise in broadly cross-reactive adaptive immunity against human β-coronaviruses in MERS-recovered patients during the COVID-19 pandemic
Source: Sci Adv. 2024 Feb 28;10(9):eadk6425. doi: 10.1126/sciadv.adk6425 (PMC10901372; doi:10.1126/sciadv.adk6425)
Supplement: Supplementary file 1 — Supplementary Materials and Methods Figs. S1 to S8 Tables S1 and S2 [file sciadv.adk6425_sm.pdf]

Science Advances
AAAS

Supplementary Materials for

Rise in broadly cross-reactive adaptive immunity against human β-coronaviruses in MERS-recovered patients during the COVID-19 pandemic

So-Hee Kim *et al.*

Corresponding author: Nam-Hyuk Cho, chonh@snu.ac.kr; Dong-Gyun Lim, limdg6761@gmail.com; Yeon-Sook Kim, idalicekim@gmail.com; Seungtaek Kim, seungtaek.kim@ip-korea.org

Sci. Adv. **10**, eadk6425 (2024)
DOI: 10.1126/sciadv.adk6425

This PDF file includes:

Supplementary Materials and Methods
Figs. S1 to S8
Tables S1 and S2

Supplementary MATERIALS AND METHODS

Enzyme-linked immunosorbent assay

MERS-CoV spike (S1 domain)-specific IgG levels were determined using semi-quantitative enzyme-linked immunosorbent assay (ELISA) using an anti-MERS-CoV S1 ELISA kit (EUROIMMUN, Lubeck, Germany), according to the manufacturer's instructions. OD ratios were calculated by comparing the extinction values of the samples with those of the calibrator. OD ratios < 0.7, 0.7–1.4, and > 1.4 were considered negative, intermediate, and positive, respectively.

To determine the antigen-specific antibody titers, His-tagged proteins were purchased from Sino Biological (Beijing, China) (hCoV-OC43 spike, catalog #:40607-V08B ; hCoV-NL63 spike, 40604-V08B ; SARS-CoV-1 Spike, 40634-V08B ; MERS-CoV Spike, 40069-V08B ; MERS-CoV S2, 40070-V08B ; SARS-CoV-2 Spike, 40589-V08B1 ; SARS-CoV-2 S2, 40590-V08B). Immunoassay plates (96-well plates; Nunc, Rochester, NY, USA) were coated with 100 ng/well of each antigen at 4°C overnight. Next, immunoassay plates were washed with phosphate buffered saline (PBS) containing 0.05% Tween 20 (PBST; Sigma-Aldrich, St. Louis, MO, USA) and blocked for 2 h at room temperature with PBST containing 5% skim milk (BD Difco, Sparks, MD, USA). Four-fold dilution with six points starting at 1:100, if not indicated otherwise, was performed for all human serum samples, and 100 μL of serially diluted serum samples were incubated for 1 h at room temperature. Subsequently, diluted (1:10,000) horseradish peroxide-conjugated anti-human IgG (H + L) antibodies (Promega, Madison, WI, USA) were used for detection. After washing the wells with 0.05% PBST, 3′,5,5′-tetramethylbenzidine peroxidase substrate solution (BioLegend, San Diego, CA, USA) was added for 5 min for color development, and the reaction was arrested by adding 1 M

$H3PO4$ solution. The absorbance was measured at 450 nm using a microplate reader (TECAN, Mannedorf, Switzerland). Sera from healthy controls unexposed to MERS-CoV and SARS-CoV-2 antigens were used to calculate the cutoff values (mean + 2 × SD) in every assay plate.

ELISA was performed, to detect influenza A virus-specific IgG levels, using an anti-influenza virus A IgG ELISA kit (Mybiosource, Vancouver, BC, Canada), according to the manufacturer's instructions. The assay plate was coated with inactivated influenza Virus A antigens. Units (U) were calculated by dividing the 10 × sample absorbance values by the cutoff values. Results below and above 11 U were considered negative and positive, respectively. To examine the levels of rhinovirus-specific IgG, ELISA was performed using an anti-human rhinovirus IgG ELISA kit (Abbexa, Cambridge, UK), according to the manufacturer's instructions. The assay plate was coated with inactivated rhinovirus antigens. The cutoff value was calculated by adding 0.15 to the mean negative control OD value. Mean sample OD values greater and lesser than the cutoff value were considered positive and negative, respectively.

ELISA was performed using peptides as previously described (*41*). The N-terminal biotinylated peptide (PLLGNSTGIDFQDELDEFFKNVSTSIP derived from CE#4) was synthesized by Synpeptide Co., Ltd. (Shanghai, China). Streptavidin (2 μg/mL in PBS, Thermofisher) was coated on 96-well plates (Thermofisher) overnight at 4°C. After washing thrice with 0.05% PBST, 3% bovine serum albumin in PBST was used to block the plates for 2 h at room temperature. Further, 0.5 μg/mL N-terminal biotinylated peptide was added and incubated for 1 h at room temperature. After washing thrice with 0.05% PBST, serially diluted serum samples starting at 1:20 were added to the plates and incubated for 1 h at room

temperature. After washing with 0.05% PBST, anti-human IgG conjugated with alkaline phosphatase (1/10000 dilution) (Sigma-Aldrich) was added, and the cells were incubated for 1 h at room temperature. After the final wash, phosphatase substrate (Sigma-Aldrich) was added to each well, and then 3 N NaOH was used to arrest the reaction after 7 min. Absorption was measured at 405 nm. Sera from healthy controls unexposed to MERS-CoV or SARS-CoV-2 antigens were used to calculate the cutoff values (mean + 2 × SD) in every assay plate.

Neutralizing antibody assay

To assess the nAb titers against MERS-CoV and SARS-CoV-2 in the collected sera, an FRNT assay was performed, as described previously (*19*). For MERS-CoV, serially diluted sera, starting at 1:20, were incubated with wild-type MERS-CoV (0.0004 multiplicity of infection) isolated from a Korean patient (NCBI genome sequence: KT029139.1) for 1 h at 37 °C. The mixtures were then added in duplicate to a 24-well plate containing a monolayer of Vero E6 cells. After incubation for 1 h at 37 °C, each well was washed and overlaid with semisolid medium containing 1% methylcellulose and 10% fetal bovine serum (FBS). After 3 d of incubation, the cells were fixed with 4% paraformaldehyde. MERS-CoV foci were immunostained with rabbit anti-MERS-CoV N protein antibody (Sino Biological Inc., Beijing, China) and goat anti-rabbit IgG secondary antibody conjugated with alkaline phosphatase (Invitrogen), and visualized using nitroblue tetrazolium/5-bromo-4-chloro-3-indolyl phosphate (Merck). The percentage foci reduction was calculated using the following formula: [(number of plaques without antibody) × (number of plaques with antibody)]/(number of plaques without antibody) × 100. The $FRNT_{50}$ titers were determined

using nonlinear regression analysis via the log(inhibitor) versus normalized response method embedded in GraphPad Prism Software v8.0 (GraphPad Software Inc., San Diego, CA, USA).

For SARS-CoV-2, overlay medium containing 0.8% methylcellulose and 10% FBS was used. Both, the wild-type (NCBI genome sequence: MW466791.1, NCCP no. 43326) and Omicron BA.5 variant (NCCP no. 43426) SARS-CoV-2 isolated from Korean patients, were kindly provided by the Korea Centers for Disease Control and Prevention (KCDC, Osong, Republic of Korea). All experiments using MERS-CoV and SARS-CoV-2 were performed in the Biosafety Level 3 (BSL-3) laboratory at Seoul National University College of Medicine.

Microneutralization assay

To assess the serum nAb titers against SARS-CoV-1, a microneutralization assay was used. Vero cells were obtained from the American Type Culture Collection (ATCC CCL-81) and maintained at 37 °C with 5% CO2 in Dulbecco's Modified Eagle's Medium (DMEM, Welgene, Gyeongsangbuk-do, South Korea), supplemented with 10% heat-inactivated FBS and 1× antibiotic-antimycotic solution (Gibco). SARS-CoV-1 (HKU-39849) was provided by Dr. Malik Peiris (Hong Kong University). For measuring serum nAb levels, Vero cells were seeded at 0.5×10^4 cells per well in DMEM supplemented with 2% FBS and 1 × antibiotic-antimycotic solution (Gibco) in flat-bottom 96 microwell plates (Thermo Scientific) 24 h prior to the experiment. Sera were diluted to an initial dilution of 1:2, followed by 1:3 serial dilutions in PBS (Welgene). The diluted sera were pre-incubated for 30 min at 37 °C with 100 × 50% tissue culture infective dose (100× $TCID_{50}$) of SARS-CoV-1 in a 1:1 ratio. Then the mixture was added to a monolayer of Vero cells in a 96-well plate and incubated for 4

days at 37 °C in 5% CO2. The nAb titer of each sample was assessed by microscopically observing the virus-induced cytopathic effects, and the inhibitory dilution factor at 50% neutralization was calculated using a non-linear regression curve fit model using Prism 6 software (GraphPad Software). An anti-SARS-CoV-1 spike antibody (Sino Biological) was used as a reference nAb. The experiment was conducted in quadruplicate, and all experiments using SARS-CoV-1 were performed in a Biosafety Level 3 (BSL-3) laboratory at Institut Pasteur, Korea.

Peptide microarray

Peptide microarray analysis was performed by PEPperPRINT GmbH (Heidelberg, Germany), using the PEPperCHIP® Pan-Corona Spike Protein Microarray and PEPperMAP® Single Isotype Assay Service. These pan-corona spike protein microarrays contained seven spike peptide libraries from SARS-CoV-2 (UniProt ID: P0DTC2), SARS-CoV (UniProt ID: P59594), MERS-CoV (UniProt ID: A0A140AYZ5), HCoVOC43 (UniProt ID: P36334), HCoV-HKU1 (UniProt ID: U3NAI2), and HCoVNL63 (UniProt ID: Q6Q1S2) and HCoV-229E (UniProt ID: P15423) as well as all spike protein mutations of the SARS-CoV-2 Alpha, Beta, Gamma, Delta, Delta Plus, and Omicron variants of concern (sequence information is available in https://www.pepperprint.com/). These spike protein sequences were translated into 4,979 linear 15 AA peptides printed in duplicate (9,958 peptide spots), with a peptide overlap of 13 AA. Each microarray contained HA (YPYDVPDYAG, 114 spots) and polio (KEVPALTAVETGAT, 114 spots) epitopes as control peptides.

Cell preparation

PBMCs were isolated using Ficoll-Paque (GE Healthcare, Sweden) density gradient centrifugation and stored in liquid nitrogen until use. Thawed PBMCs were washed twice with Roswell Park Memorial Institute (RPMI) 1640 medium supplemented with 2 mM glutamine, 1% (v/v) nonessential amino acids, 1% (v/v) sodium pyruvate, 1% (v/v) 4-(2-hydroxyethyl)-1-piperazineethanesulfonic acid (HEPES), penicillin (50 U/mL), streptomycin (50 g/mL), and 10% FBS (all from Invitrogen, USA). The cells were then rested in RPMI-CM at 37 °C and 5% CO2 overnight prior to use in ELISpot and intracellular cytokine staining assay.

Synthetic viral peptides

The genomic sequence of a representative Korean isolate of MERS-CoV (KOREA/Seoul/014-2015, NCBI accession number KT374052) was used to generate a library of peptides spanning all four MERS-CoV structural proteins as described previously (*21*). Peptides were synthesized by Mimotopes (Mulgrave, Victoria, Australia) as 15 AA long with 11 AA overlap. The purity of the peptides was > 80% as determined using mass spectrometry and high-performance liquid chromatography. The peptides were dissolved at 80 mg/mL in Dimethyl sulfoxide (DMSO) and pooled as those encompassing the viral S protein-containing set into two sets (S1:168 N-terminal peptides; S2:168 C-terminal peptides) and the E, M, and N proteins-containing set (E/M/N: 171 peptides).

IFN-γ ELISpot assay

ELISpot assay was performed using a commercially available kit (Mabtech, Nacka Strand, Sweden) according to the manufacturer's instructions. The PBMCs were plated at 2×10^5 cells/well and incubated for 21–23 h in 200 μL RPMI-CM under stimulation with three pools of synthetic viral peptides (S1, S2, and E/M/N; 1 μg/mL of each peptide). The positive control was anti-CD3 (1 μg/mL, BD Biosciences), and the negative control was DMSO at a concentration equivalent to that of the peptide pools. Spots were counted using a CTL ELISpot reader (CTL Analyzer, Shaker Heights, OH, USA). The overall response to MERS-CoV antigens was defined as the sum of the background (without antigens)-subtracted response to each pool. A sample was considered positive if any of the T cell responses to individual peptide pool stimulations were positive. Positive results were defined as the number of SFCs higher than the mean value plus two SD observed in control samples from uninfected healthy donors, as previously described (*21*).

Intracellular cytokine staining and flow cytometric analysis

Thawed and rested PBMCs were stimulated for 1 h with three pools of synthetic viral peptides (S1, S2, and E/M/N; 1 μg/mL of each peptide) in the presence of 1 μg/mL anti-CD28 and anti-CD49d monoclonal Abs (BD Bioscience). For negative and positive control cultures, cells were incubated with DMSO alone and anti-CD3 monoclonal antibody, respectively. After adding 1 μg/mL Brefeldin A (eBiosciences) and 0.7 μg/mL Monensin (BD Biosciences), the cells were incubated for an additional 5 h. Following stimulation, the cells were surface-stained with Aqua Dead Cell Stain Kit (Molecular Probes) and then a mixture of mAbs containing anti-CD3 BV421, anti-CD4 PerCP-Cy5.5, and anti-CD8 APC-H7 (all from BD Biosciences). After fixation and permeabilization using the Intracellular Fix

and Perm Set (eBiosciences), the cells were stained with anti-IFN-γ PE-Cy7 (eBiosciences), anti-IL-2 PE (eBiosciences), and anti-TNF-APC (BD Biosciences) mAbs. After labeling, the cells were resuspended with PBS containing 1% paraformaldehyde and stored at 4 °C prior to flow cytometry analysis within 24 h. At least 100,000 stained cells were acquired per sample using a FACSverse Flow Cytometer (BD Sciences) and analyzed using FlowJo software (Tree Star, Ashland, OR, USA). The gating strategy is presented in fig. S8. The data presented correspond to background-subtracted results using a negative control culture, and values > 0.01% and > 2 × the background were considered positive.

Bio-informatic analysis

Unsupervised sample clustering was performed using the k-means algorithm. The optimum number of clusters was determined through silhouette coefficient analysis using the NBClust and Factoextra packages in R (version 1.0.7). Prior to clustering and visualization, each feature was standardized using z-score transformation with the scale function of the R software (version 4.1.0). The Complex Heatmap R package (version 2.10.0) (*49*) was used to generate a heat map for visualization.

Statistical analyses

Statistical analyses of different groups were performed using a two-tailed Mann–Whitney test or nonparametric one-way analysis of variance, followed by the Kruskal–Wallis test for multiple comparison. Correlations between variables were assessed using the Spearman's

correlation test. $p < 0.05$ was considered statistically significant. All data analyses were performed using the GraphPad Prism Software.

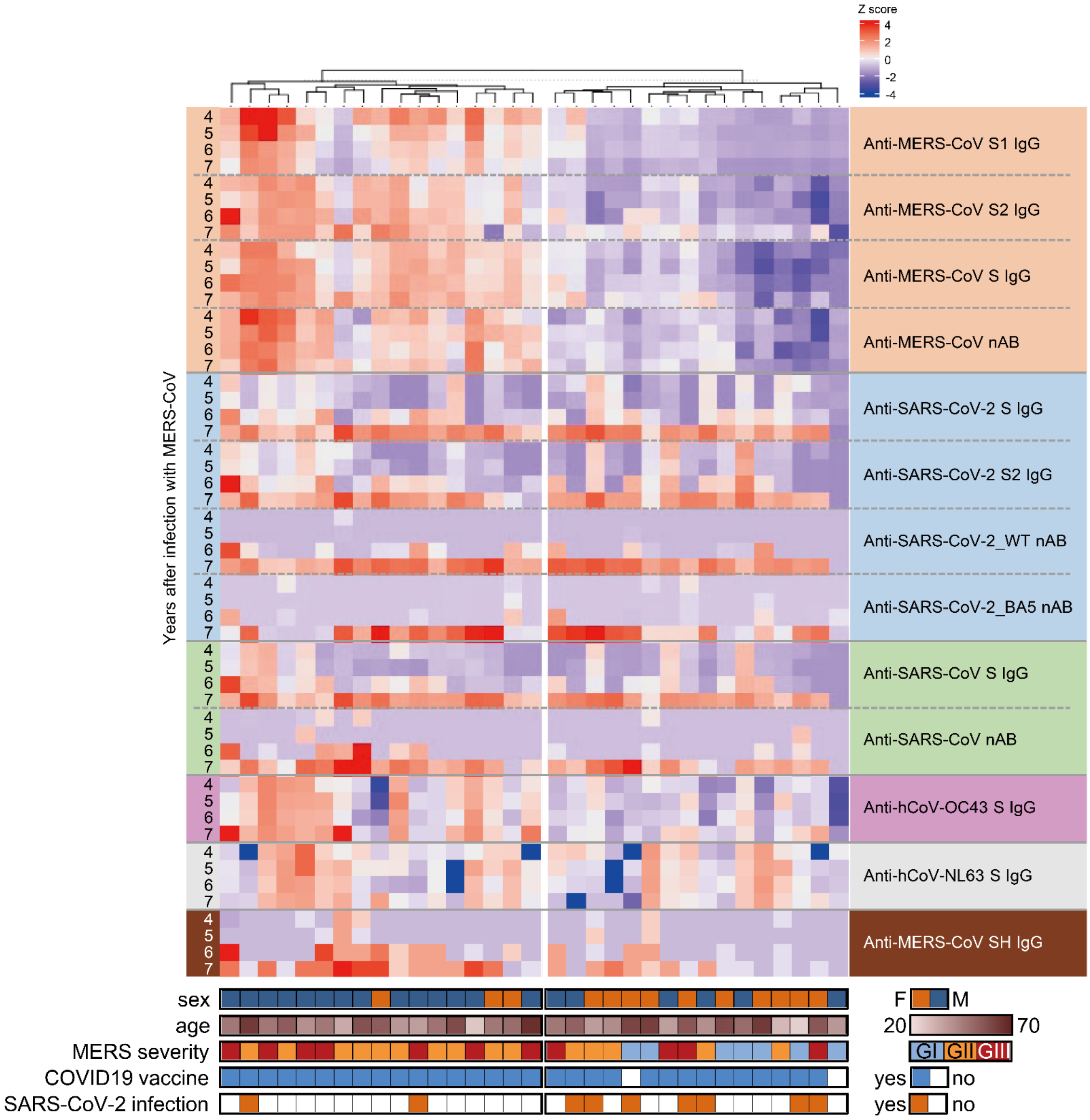

fig. S1. Unsupervised hierarchical clustering of MERS survivors based on the levels of antibodies and neutralizing activity. The antibody levels are arranged for the collection year after the 2015 MERS outbreak (y-axis). Heatmap shows normalized z-scores within each antibody level, and clustering was determined using correlation and average linkage in all datasets. Baseline characteristics of the participants are included in the bottom panels.

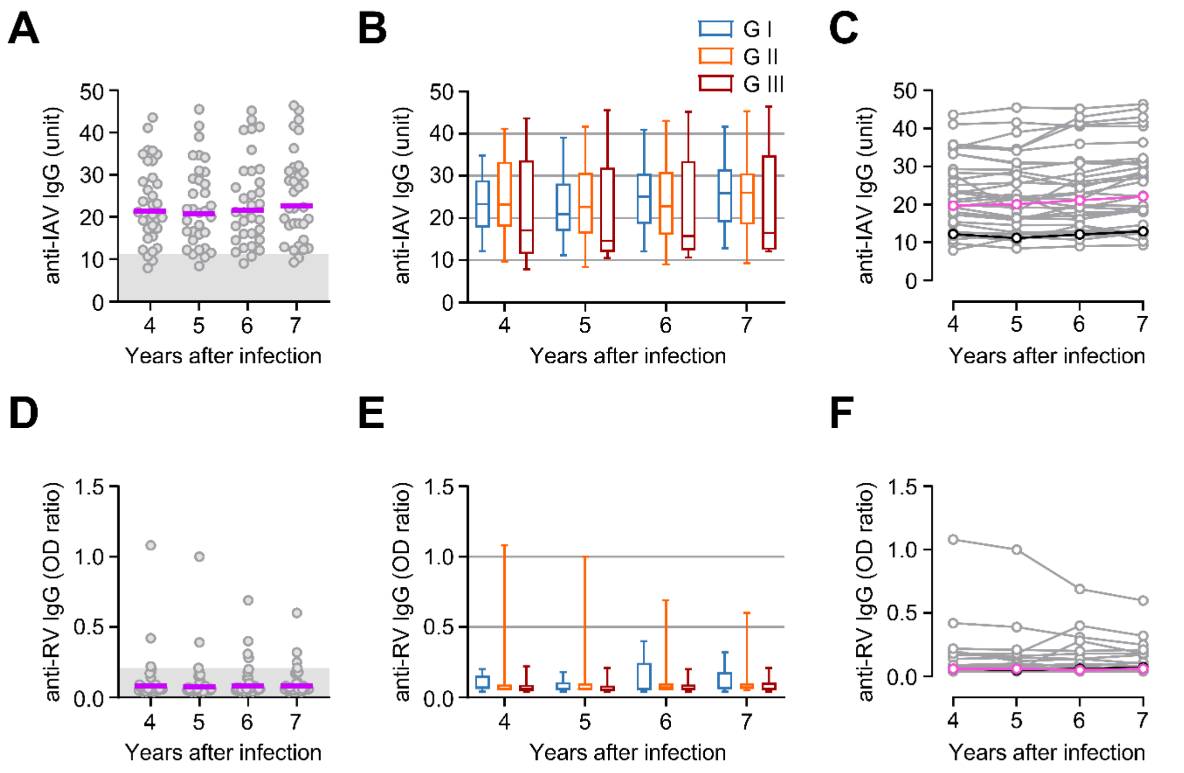

fig. S2. Kinetic changes of antibody responses against influenza A virus and rhinovirus. (**A** and **D**) The antibody response levels against human influenza A virus (IAV) (**A**) and rhinovirus (RV) (**D**) in 33 subjects whose sera were available from the fourth to the seventh year after the Korean MERS outbreak. Purple line: geometric mean; gray box: cut-off value zone. (**B** and **E**) The antibody response levels against human influenza A virus (**B**) and rhinovirus (**E**) are presented according to clinical MERS severity. (**C** and **F**) Kinetic changes in the levels of antibodies against human influenza A virus (**C**) and rhinovirus (**F**) of individual participants are presented. The antibody response levels of the participant who was confirmed to have SARS-CoV-2 infection without COVID-19 vaccination are colored in pink, while those of the another one without confirmed COVID-19 infection and vaccination are colored in black.

A

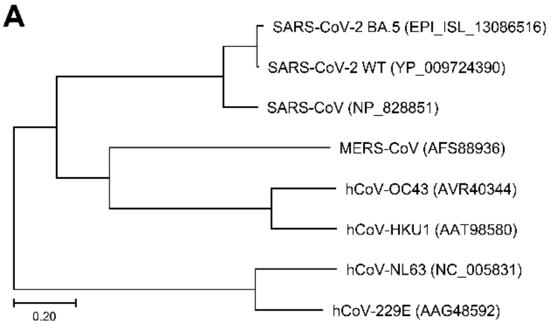

B

		SARS-CoV-2 WT	SARS-CoV-2 BA.5	SARS-CoV	MERS-CoV	hCoV-OC43	hCoV-HKU1	hCoV-NL63	hCoV-229E
S	SARS-CoV-2 WT		97.3	76.0	31.1	29.6	28.7	24.9	26.5
	SARS-CoV-2 BA.5	97.8		75.2	30.2	29.6	28.6	24.9	25.9
	SARS-CoV	87.1	87.1		30.5	29.9	28.6	23.6	25.8
	MERS-CoV	48.0	47.4	47.1		31.8	31.1	25.3	25.6
	hCoV-OC43	45.8	45.4	46.3	50.1		62.8	25.7	24.9
	hCoV-HKU1	47.1	46.8	46.5	50.4	78.7		24.3	24.7
	hCoV-NL63	42.0	42.2	41.7	43.4	43.4	41.4		55.6
	hCoV-229E	428.	42.5	43.3	40.4	40.1	41.1	67.9	
S1	SARS-CoV-2 WT		95.6	64.3	22.3	21.4	20.8	18.2	19.7
	SARS-CoV-2 BA.5	96.5		62.9	21.2	21.5	20.6	18.3	18.8
	SARS-CoV	78.7	78.8		21.7	22.6	22.4	16.7	18.1
	MERS-CoV	38.3	36.6	36.6		22.3	22.4	15.4	17.2
	hCoV-OC43	36.9	36.3	38.0	42.0		54.7	17.8	18.3
	hCoV-HKU1	38.7	38.1	38.9	43.8	72.8		16.9	16.8
	hCoV-NL63	34.6	34.3	33.1	30.8	34.3	32.2		37.0
	hCoV-229E	32.8	32.5	32.7	29.8	31.9	32.3	51.9	
S2	SARS-CoV-2 WT		99.3	90.0	42.1	40.3	38.4	31.6	33.1
	SARS-CoV-2 BA.5	99.3		89.6	41.9	40.0	38.4	31.3	32.7
	SARS-CoV	96.9	96.6		41.6	39.7	37.2	30.7	33.4
	MERS-CoV	60.0	60.0	59.8		45.0	42.3	34.8	34.2
	hCoV-OC43	58.0	57.8	57.0	60.6		72.9	34.4	32.5
	hCoV-HKU1	57.7	58.1	55.9	57.6	84.9		32.9	33.1
	hCoV-NL63	50.2	50.1	50.2	51.3	50.9	50.7		70.4
	hCoV-229E	52.5	52.3	52.5	51.8	49.1	51.0	80.0	

Similarity (%) (lower triangle); Identity (%) (upper triangle)

fig. S3. Phylogenetic analysis of seven hCoVs. (**A**) Phylogenetic tree of seven hCoVs generated by amino acid sequence alignment using the MUSCLE algorithm and the maximum likelihood method. Scale bar, 0.20 substitutions per amino acid site. (**B**) Similarity and identity of amino acid sequences are computed using the BLOSUM62 matrix.

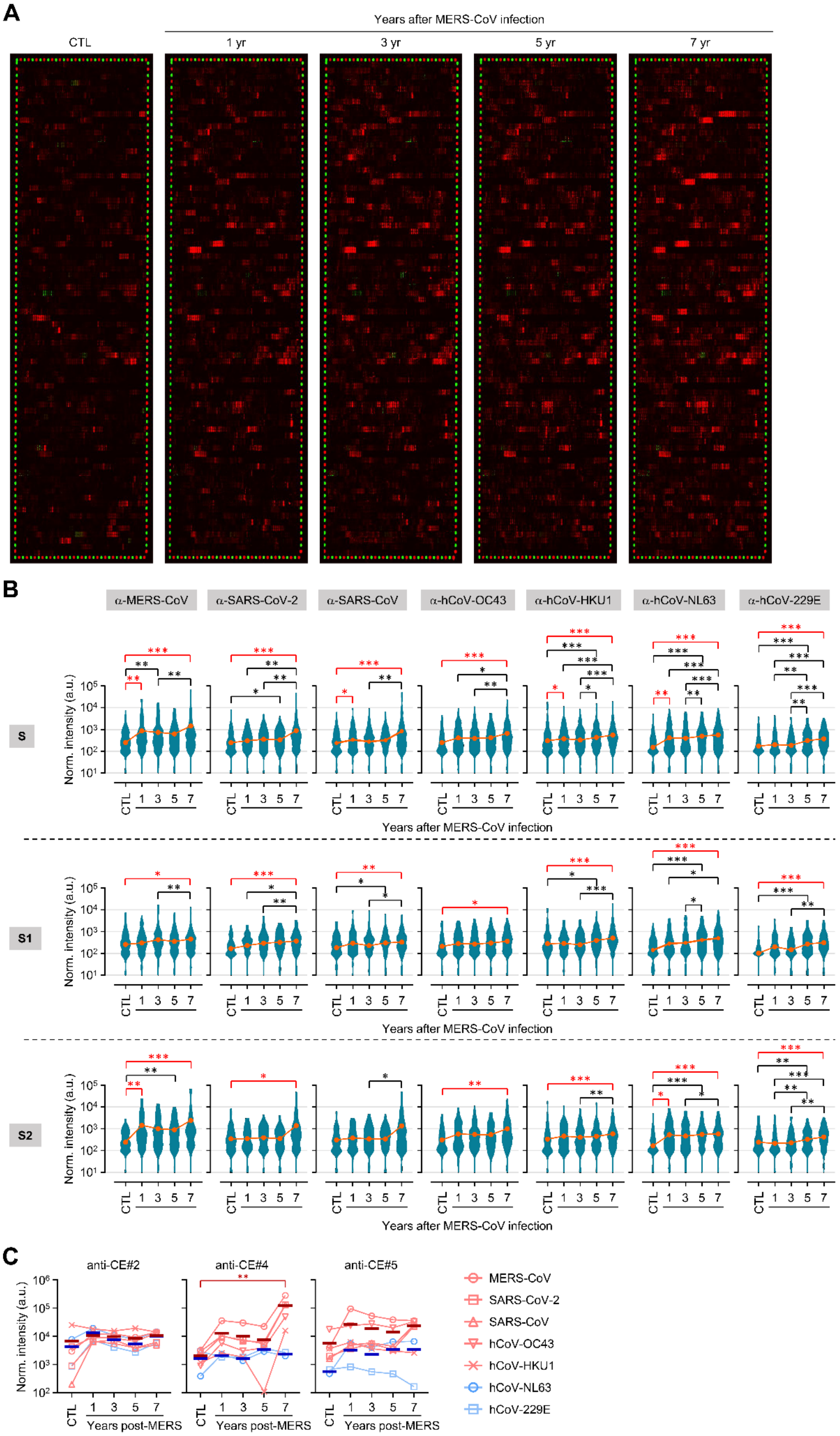
A
Years after MERS-CoV infection
CTL
1 yr
3 yr
5 yr
7 yr
B
α-MERS-CoV
α-SARS-CoV-2
α-SARS-CoV
α-hCoV-OC43
α-hCoV-HKU1
α-hCoV-NL63
α-hCoV-229E
S
S1
S2
Norm. intensity (a.u.)
CTL 1 3 5 7
Years after MERS-CoV infection
C
anti-CE#2
anti-CE#4
anti-CE#5
Norm. intensity (a.u.)
Years post-MERS
MERS-CoV
SARS-CoV-2
SARS-CoV
hCoV-OC43
hCoV-HKU1
hCoV-NL63
hCoV-229E

fig. S4. Kinetic landscapes of antibody responses against overlapping 15-mer peptides derived from various hCoVs' spikes. (**A**) Pooled sera from recovered MERS patients collected at the indicated time were applied for antibody reactivity assay using pan-CoV spike protein microarray. Fluorescence images of the results are presented. CTL: negative control pooled sera. (**B**) The sum of antibody responses against overlapping linear peptides derived from indicated spike antigens is presented. Normalized intensity data indicated here were generated from (**A**). Orange dot: mean intensity. *, $p < 0.05$; **, $p < 0.01$; ***, $p < 0.001$ by Kruskal–Wallis test. (**C**) Kinetic changes in antibody levels against indicated viral spike epitopes (pink: anti-βCoVs antibodies, brown line: mean of anti-βCoVs antibodies, sky blue: anti-αCoVs antibodies, indigo line: mean of anti-αCoVs antibody). **, $p < 0.01$ (among βCoV group) by Kruskal–Wallis test.

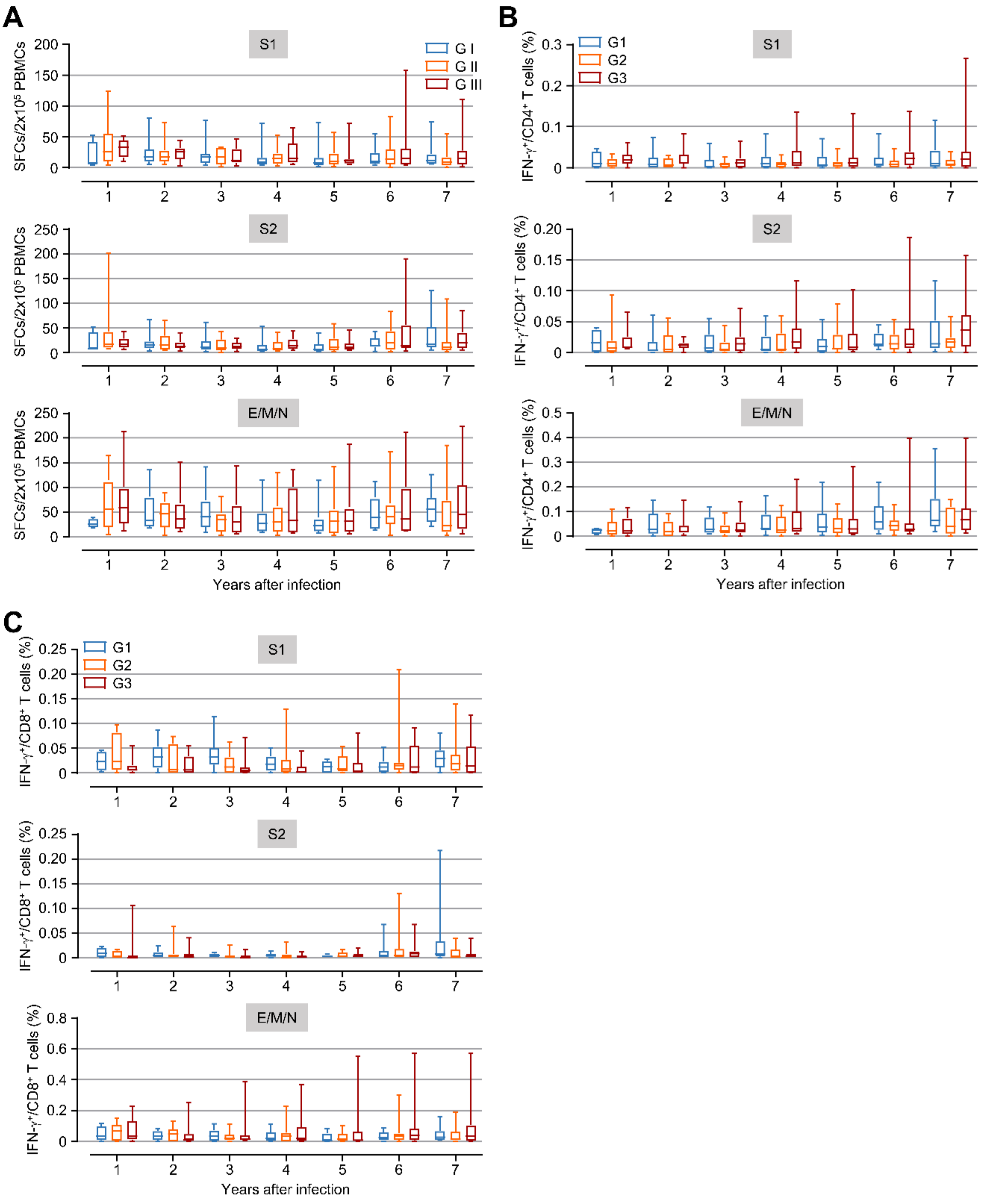

fig. S5. Kinetic changes of antiviral T cell responses, according to clinical MERS severity, in MERS survivors. (**A**) Kinetic changes in memory T cell responses against the indicated structural antigens were measured using IFN-γ ELISpot assays and are presented according to clinical MERS severity. Box and whisker (min to max) plots, including the median value. Sample numbers/year are indicated in Fig. 7A. (**B** and **C**), Kinetic changes in memory CD4$^+$ T

cell (**B**) and CD8$^+$ T cell (**C**) responses against the indicated structural antigens were measured using flow cytometry and are presented according to clinical MERS severity. Box and whisker (min to max) plots, including the median value.

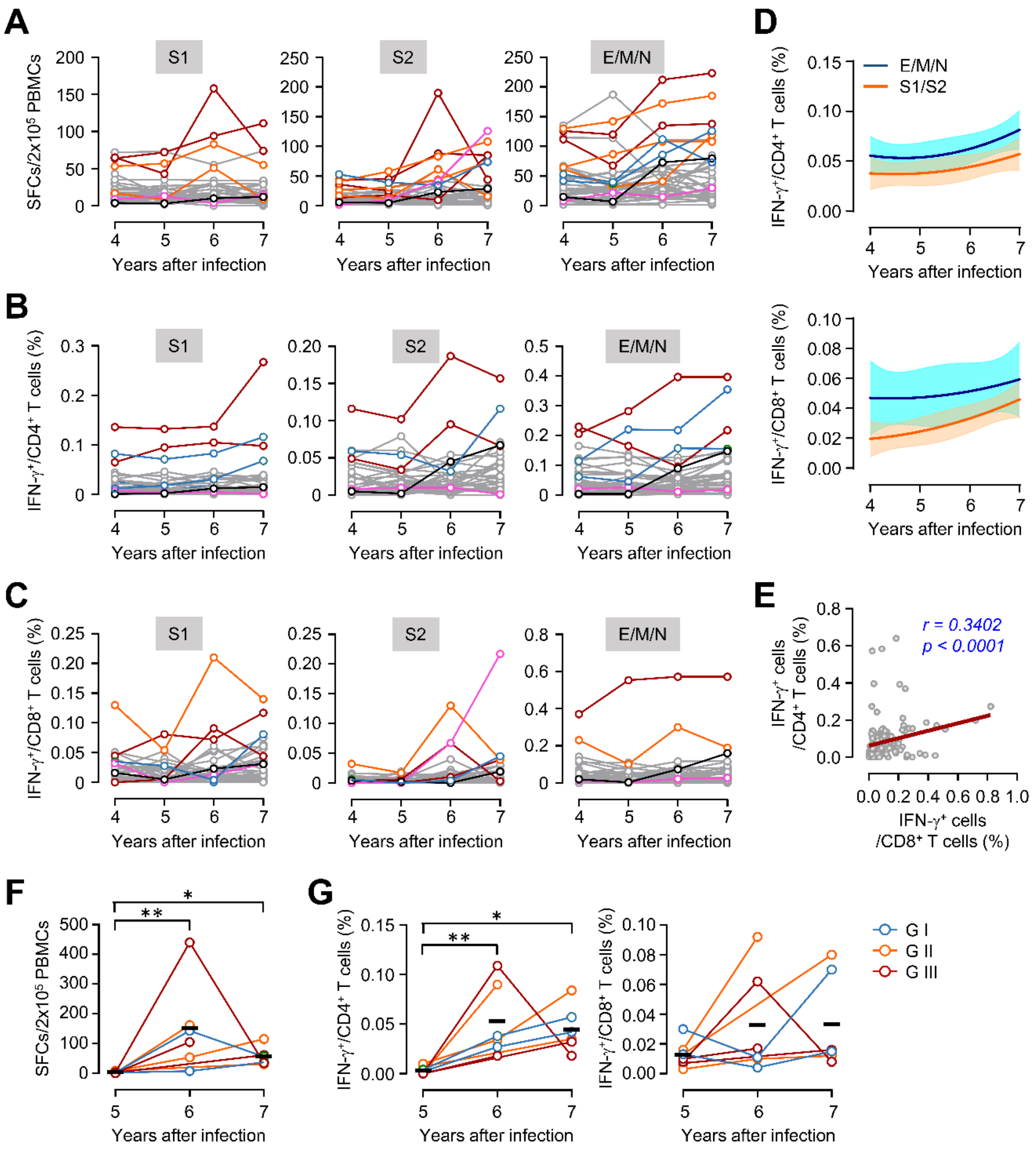

fig. S6. Kinetic changes in antiviral T cell responses in MERS survivors. (**A-C**) Memory T cell response kinetics to indicated peptide antigen pools, measured using ELISpot assay (**A**) and flow cytometry (**B**, CD4$^+$ T cells; **C**, CD8$^+$ T cells). Kinetics in participants with more than 1.5 times increase in memory T cell responses during the COVID-19 pandemic compared to those before the pandemic, according to MERS severity (light blue: G I, orange: G II, brown: G III, pink: unvaccinated and SARS-CoV-2-infected participant, black: unvaccinated and uninfected participant). *n* = 33/year. (**D**) Mean memory T cell response kinetics after non-linear

regression analysis with 95% CI. (**E**) Correlation of IFN-γ^+ $CD4^+$ T cells with IFN-γ^+ $CD8^+$ T cells responding to the structural peptide pools assessed using linear regression (brown line) and Spearman's rank test. $n = 132$. (**F** and **G**) Kinetic changes in T cell responses against the SARS-CoV-2 structural antigens (S1/S2/E/M/N) were measured using IFN-γ ELISpot assays (**F**) and using flow cytometry (**G**) on PBMCs collected at the indicated time. Individual responses ($n = 8$) before (the fifth year) and after (the sixth and seventh years) the COVID-19 pandemic are colored according to the MERS severity. Black line: mean. *, $p < 0.05$; **, $p < 0.01$ by Kruskal–Wallis test.

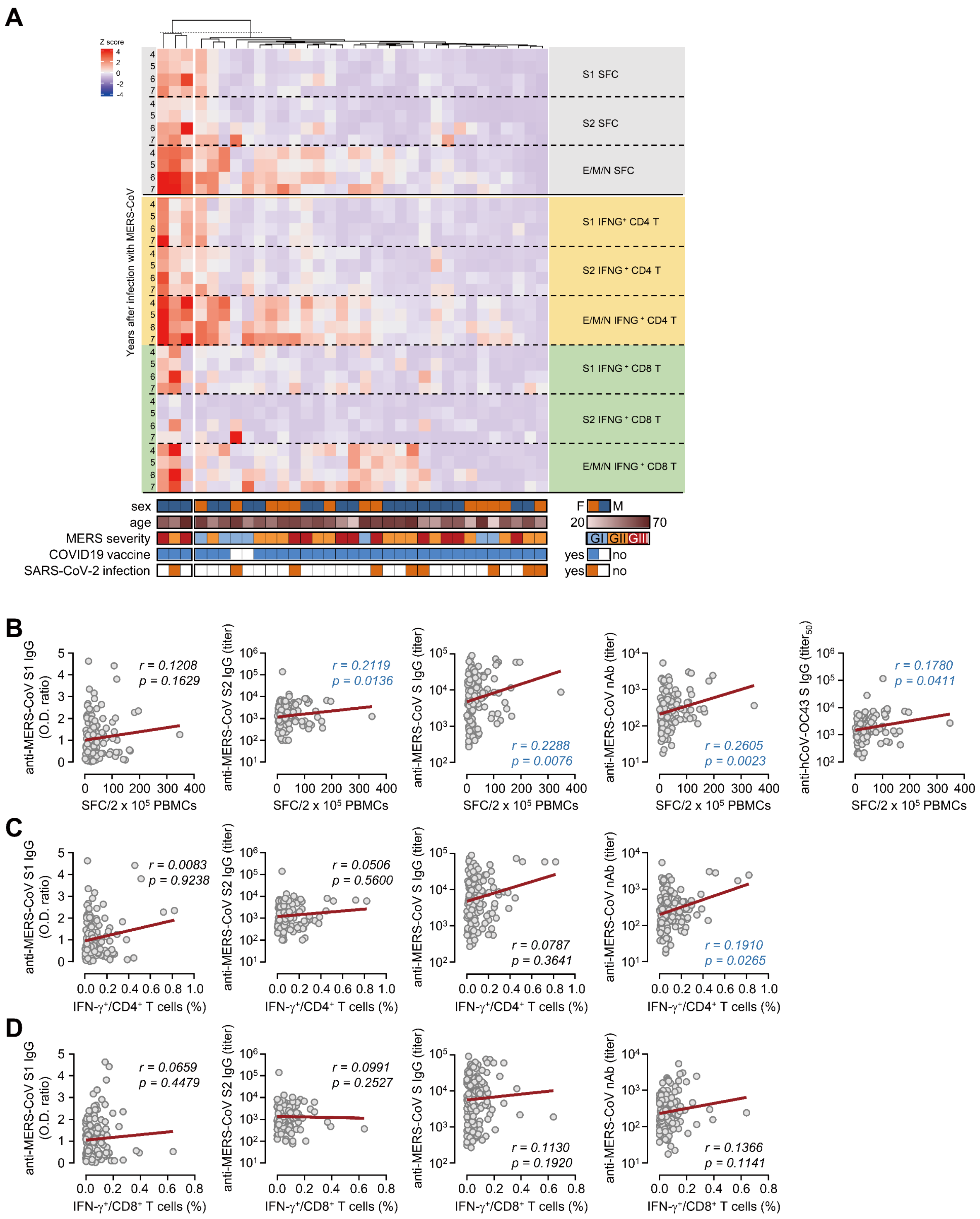

fig. S7. Unsupervised hierarchical clustering of MERS survivors based on the levels of memory T cell responses and their correlation with antibody levels to hCoVs. (**A**) The memory T cell levels specific to the indicated structural antigens of MERS-CoV are arranged according to collection year after the 2015 MERS outbreak (y-axis). Heatmap shows

normalized z scores in memory T cell response measured using IFN-γ ELISpot assays (top panels) and flow cytometry (bottom panels). Unsupervised clustering was determined using correlation and average linkage in all datasets. Baseline characteristics of the participants are included in the bottom panels. SFC: spot-forming cells. (**B**) Correlation of antibody responses against indicated spike antigens and neutralizing activity against MERS-CoV with memory T cells specific to MERS-CoV's spike peptide pools (S1/S2). (**C** and **D**) Correlation of antibody responses against indicated spike antigens and neutralizing activity against MERS-CoV with IFN-γ^+ memory $CD4^+$ T cells (**C**) and IFN-γ^+ memory $CD8^+$ T cells (**D**) specific to MERS-CoV's structural peptide pools (S1/S2/E/M/N). Correlations were assessed by linear regression (brown line) and Spearman's rank test. $n = 135$.

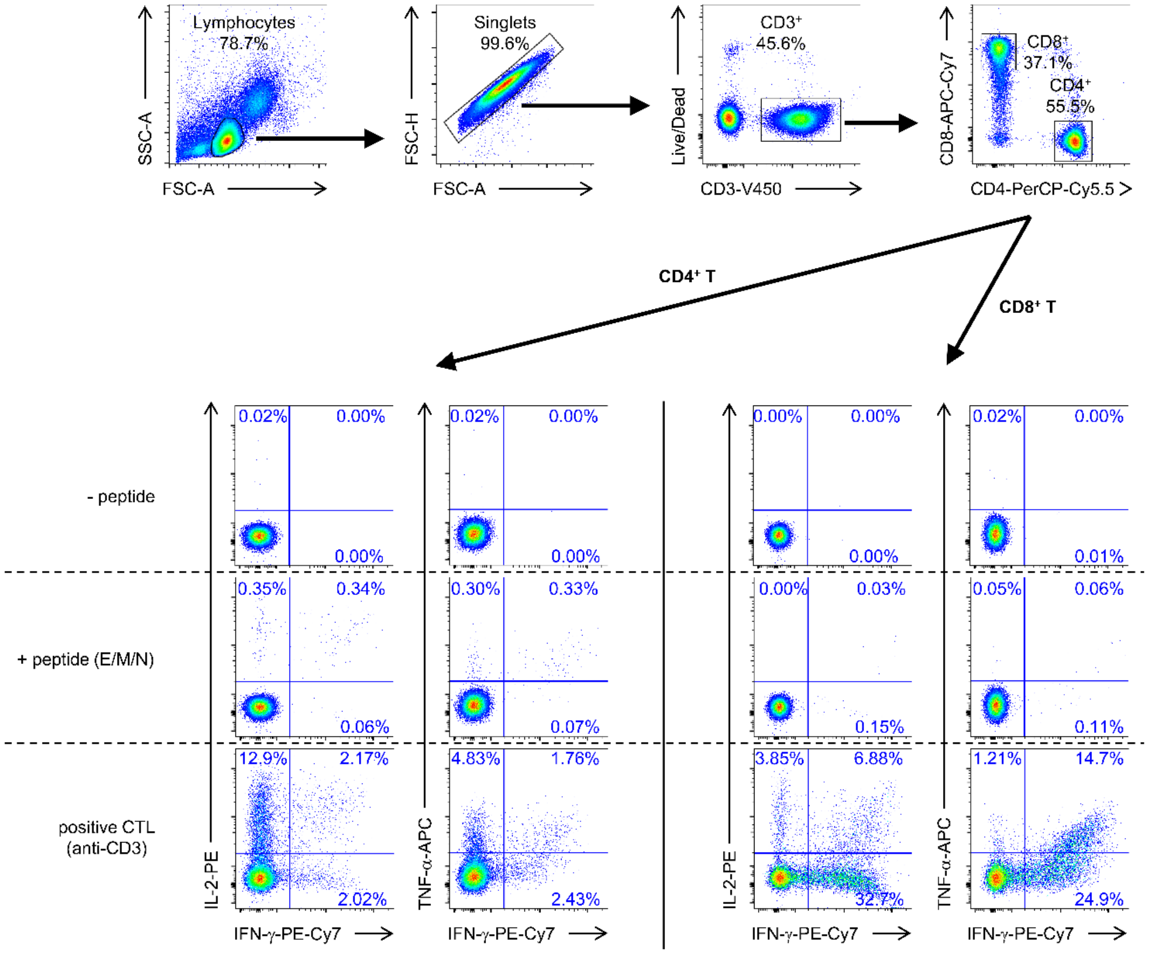

fig. S8. Gating strategy to determine the frequency of cytokine-producing CD4$^+$ or CD8$^+$ T cells. The staining profile of PBMCs from a MERS survivor after stimulation with or without MERS peptides is shown. Stimulation with anti-CD3 monoclonal antibody was used as positive control.

Table S1. Baseline characteristics of the enrolled MERS survivors and healthy controls

	MERS cohort																					Healthy controls**	COVID-19 vaccine group
MERS severity groups	Group I (G I)							Group II (G II)							Group III (G III)							NA	NA
MERS symptoms																						NA	NA
Fever duration (days, mean ± S.D.)	4.8 ± 4.9							10.9 ± 9.1							20.4 ± 11.5								
Pneumonia	No							Mild							Severe								
Oxygen treatment	No							No							Yes								
Collection time (years)	1	2	3	4	5	6	7	1	2	3	4	5	6	7	1	2	3	4	5	6	7	NA	NA
Sex (*n*)																							
Female	9	8	6	6	6	6	6	16	17	11	7	6	6	6	3	3	3	2	2	2	2	19	19
Male	9	8	5	3	3	3	3	17	15	13	13	10	9	9	15	15	15	10	11	10	9	17	20
Age (years, mean ± S.D.)*	52 (16)							49 (11)							48 (12)							52 (13)	39 (9)

***,** Data at the first collection year (2016) for MERS patients.

**, Healthy controls who were not exposed to MERS-CoV, SARS-CoV-2, and COVID-19 vaccine.

NA, not associated.

Table S2. COVID-19 vaccination and infection history of 33 participants examined for antibody tests.

I.D.	MERS severity group	Sex	Age	COVID-19 vaccine*	COVID-19 infection**	Fold change (nAb)***
1	G I	F	65	AZ-AZ-MO	-	1.24
2	G I	F	66	AZ-AZ-MO	-	0.73
3	G I	F	64	-	YES (2022)	1.22
4	G I	F	24	PF-PF	YES (2021)	2.43
5	G I	F	63	AZ-AZ-PF	-	1.71
6	G I	F	24	PF-PF	YES (2022)	0.69
7	G I	M	57	AZ-ZA-PF	-	0.76
8	G I	M	44	-	-	0.83
9	G II	M	60	AZ-AZ-PF	-	0.51
10	G II	F	57	PF-PF	-	1.15
11	G II	F	60	AZ-AZ-PF	-	0.83
12	G II	M	66	AZ-AZ-MO	YES (2022)	0.66
13	G II	M	56	AZ-AZ-MO	-	1.16
14	G II	M	62	AZ-AZ-MO	-	1.75
15	G II	M	47	MO-MO-MO	YES (2022)	1.29
16	G II	F	55	MO-MO-MO	-	0.46
17	G II	F	48	PF-PF-PF	-	1.36
18	G II	F	42	PF-PF	YES (2022)	1.17
19	G II	M	59	AZ-AZ-MO	YES (2022)	1.01
20	G II	M	44	AZ-AZ-PF	-	0.72
21	G II	M	37	AZ-PF-PF	-	2.15
22	G II	M	63	PF-PF	-	0.71
23	G II	F	32	AZ-PF-PF	-	0.78
24	G III	F	61	NV-NV	YES (2022)	0.87
25	G III	M	41	PF-PF-PF	-	1.62
26	G III	M	55	AZ-AZ-MO	-	0.70
27	G III	M	55	PF-PF-MO	-	1.49
28	G III	M	33	AZ-AZ-PF	YES (2022)	0.79
29	G III	M	68	AZ-AZ-PF	-	1.66
30	G III	M	46	PF-PF	-	1.13
31	G III	M	55	AZ-AZ-MO	-	0.64
32	G III	M	55	AZ-PF-PF	-	0.78
33	G III	M	27	PF-PF	-	1.66

*Vaccination in 2021-2022; AZ: Astrazeneca (ChAd), MO: Moderna (mRNA), PF: Pfizer (mRNA), NV: Novavax (subunit).
**Confirmed infection (infected year).
***Fold increase of nAb against MERS-CoV (mean of the sixth and seventh year/mean of the fourth and fifth year).
